# Hydration and Molar Ratio Effects in Choline Chloride-Phenol Deep Eutectic Solvents

**DOI:** 10.1021/acs.jpcb.6c01636

**Published:** 2026-05-20

**Authors:** Lucas de S. Silva, Guilherme Colherinhas

**Affiliations:** Instituto de Física, 67824 Universidade Federal de Goiás, Goiânia 74690-900, Goiás, Brazil

## Abstract

Deep eutectic solvents (DESs) based on choline chloride and phenol (CCPhe) have attracted increasing attention due to their tunable physicochemical properties and structural versatility. In this work, molecular dynamics (MD) simulations were performed for CCPhe systems at molar ratios of 1:2, 1:3, and 1:4 with water contents ranging from 0 to 30%. Energetic, hydrogen-bond, dielectric, transport, and structural analyses were combined to establish a multiscale description of hydration effects. Hydration promotes a progressive redistribution of stabilization from chloride-phenol and choline-chloride interactions toward chloride-water interactions, accompanied by increased water-mediated hydrogen bonding and reduced hydrogen-bond lifetimes. The dielectric constant increases significantly with water content, reaching 15.93 for the 1:4 system at 30% hydration, while the infinite-system Kirkwood factor reveals enhanced cooperative dipolar correlations at high hydration levels. Diffusion coefficients increase by nearly an order of magnitude between dry and highly hydrated systems, indicating substantial mobility enhancement. Radial distribution functions show that hydration modifies the first solvation shell of choline through competitive coordination between chloride and water without altering the characteristic contact distance. These results demonstrate that controlled hydration acts as an effective tuning parameter in CCPhe systems, modulating energetic balance, hydrogen-bond connectivity, collective polarization, and molecular transport in a composition-dependent manner.

## Graphical abstract

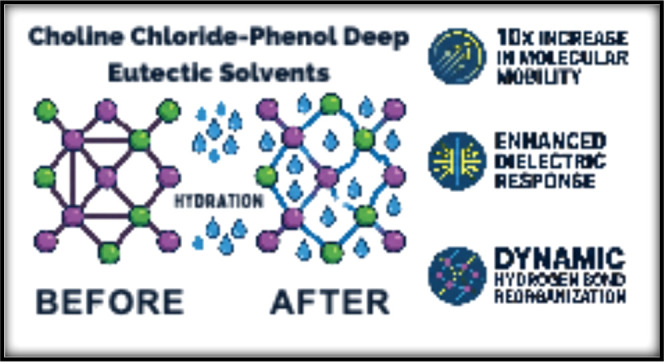

## 1 Introduction

Deep eutectic solvents (DESs) have consolidated as a versatile class of hydrogen-bonded liquids formed by combining a halide salt and a hydrogen-bond donor (HBD), typically exhibiting a marked melting-point depression relative to their pure constituents.^1^ Since the original formulation and consolidation of the DES concept, these fluids have been increasingly explored as designer solvents due to their low volatility, compositional tunability, and broad applicability across extraction, catalysis, electrochemistry, and materials processing.^2–5^ Importantly, the same structural features that enable DES formation (namely, strong directional interactions, often mediated by chloride anions in Type-III DESs, and extended H-bond networks) also control macroscopic observables such as viscosity, dielectric response, and molecular mobility, which are central for rational formulation.^1,6^ (see Figure 03)

**Figure 01:**
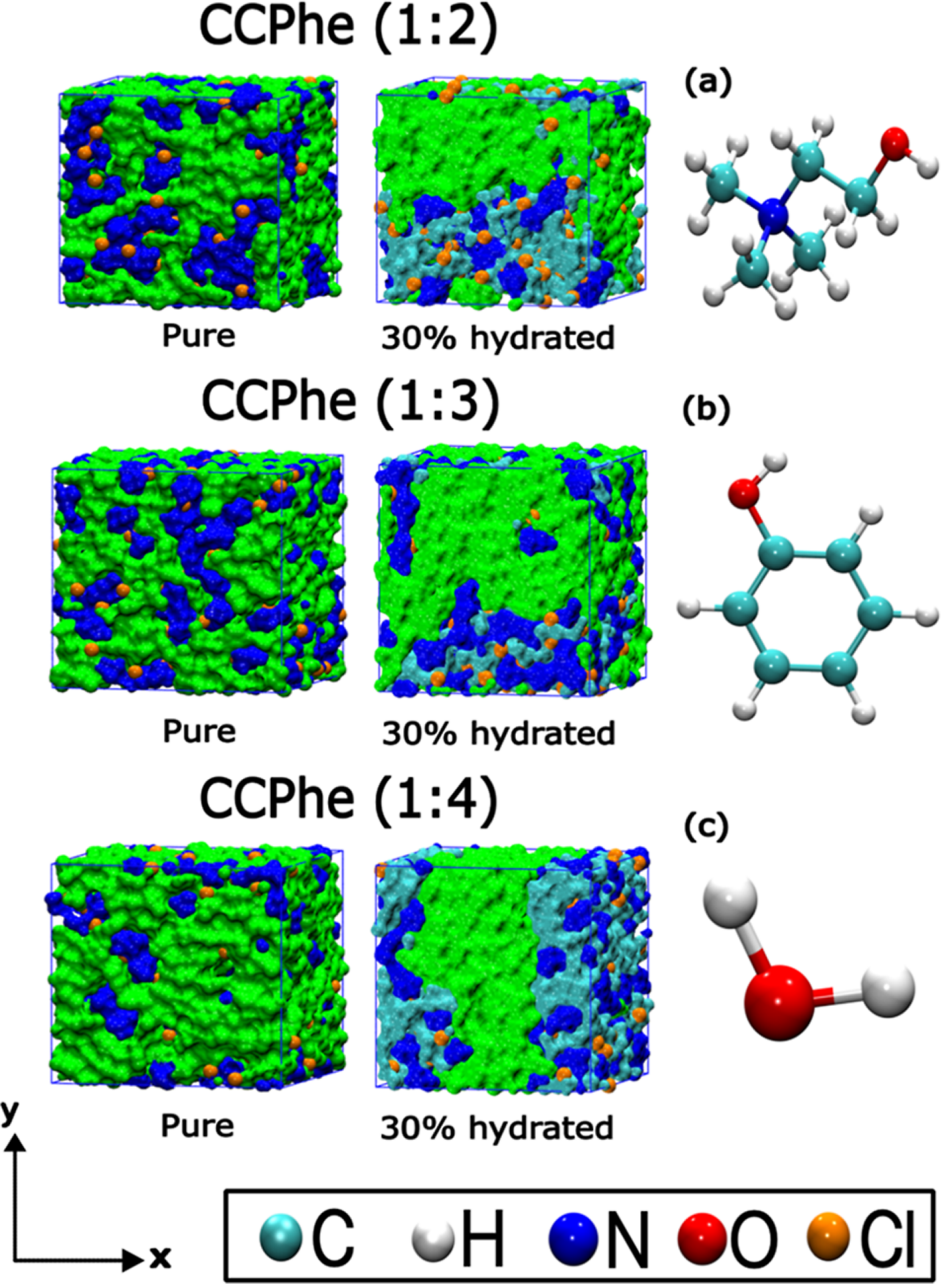
Structural visualization of representative *CCPhe* systems. Configurations corresponding to the pure DES (0% hydration) and to the highest hydration level evaluated (30%) are illustrated. The constituent molecules are depicted individually: (a) choline (*Cho*), (b) phenol (*Phe*), and (c) water, with atoms identified via a standardized color-coding scheme.

**Figure 02:**
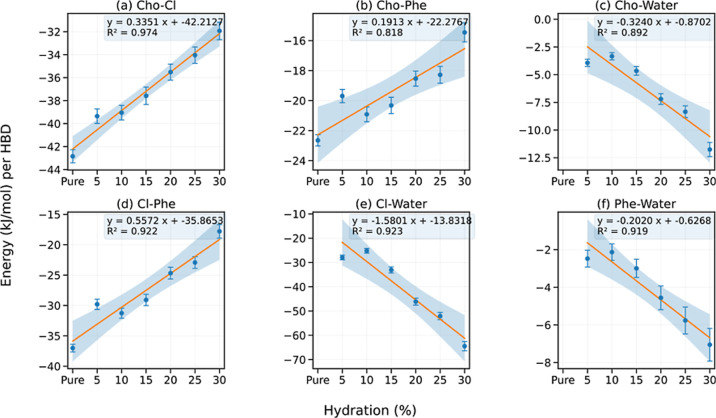
Average short-range interaction energy (Coulomb and Lennard-Jones) per HBD (kJ/mol) as a function of hydration level for the (a) *Cho*-*Cl*, (b) *Cho*-*Phe*, (c) *Cho*-Water, (d) *Cl*-*Phe*, (e) *Cl*-Water, and (f) *Phe*-Water pairs in the *CCPhe* ^1:2^ system. Symbols represent the mean values obtained from the production trajectories, with error bars. Solid lines indicate linear fits highlighting the systematic energetic redistribution induced by hydration. Increasing water content progressively weakens intrinsic DES interactions (*Cho*-*Cl*, *Cho*-*Phe*, and *Cl*-*Phe*) while strengthening chloride-water stabilization, evidencing competitive solvation and reorganization of the eutectic network.

**Figure 03:**
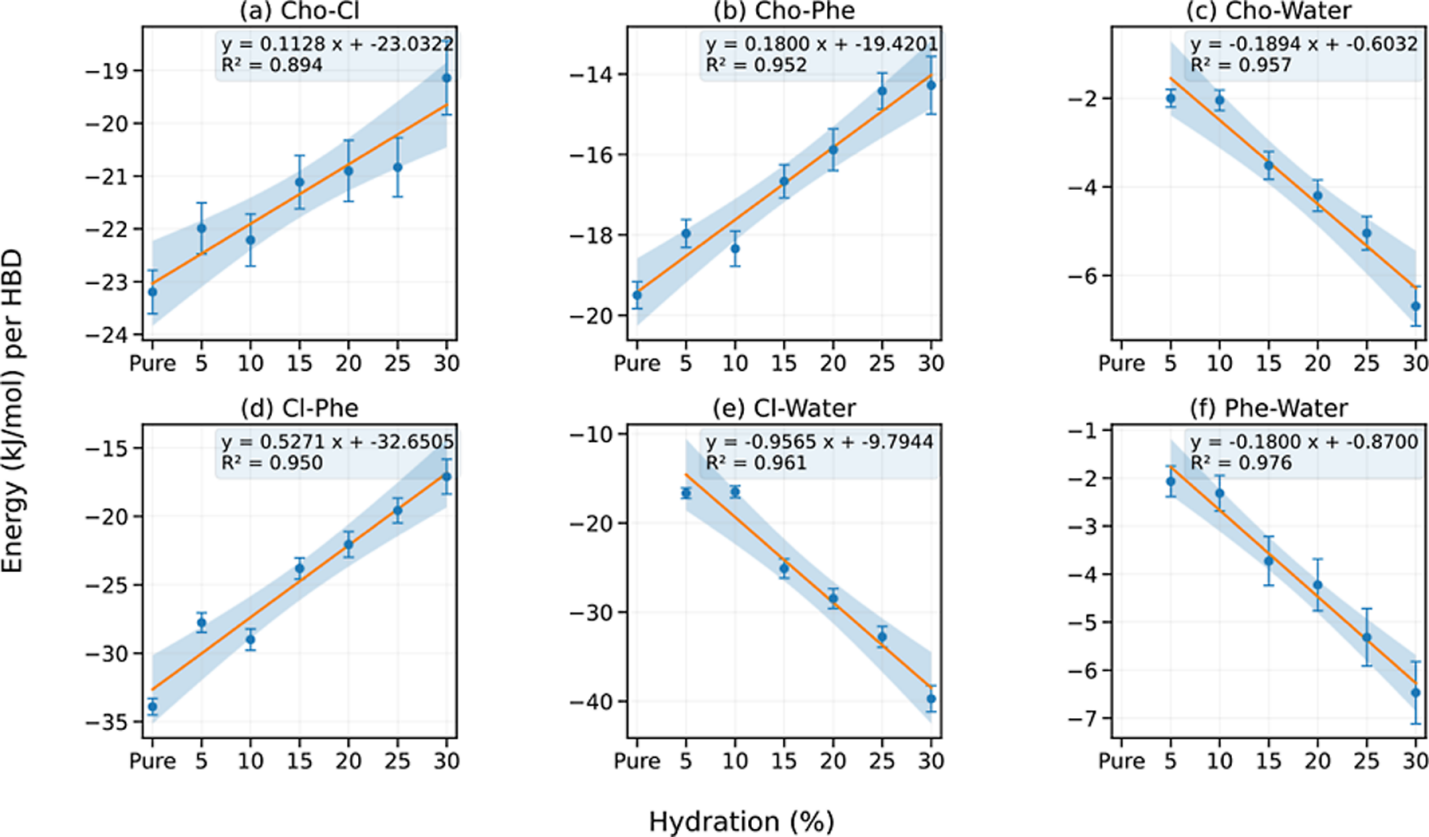
Average short-range interaction energy (Coulomb and Lennard-Jones) per HBD (kJ/mol) as a function of hydration level for the (a) *Cho*-*Cl*, (b) *Cho*-*Phe*, (c) *Cho*-Water, (d) *Cl*-*Phe*, (e) *Cl*-*Water*, and (f) *Phe*-Water pairs in the *CCPhe* ^1:3^ system. Symbols represent mean values extracted from the production trajectories, with error bars. Solid lines correspond to linear fits (*R* ^2^ values indicated in each panel) used to highlight hydration-driven energetic trends.

A recurring practical and fundamental aspect in DES research is the role of water.^7–9^ Water is frequently present as an unavoidable impurity or added intentionally to mitigate high viscosity and enhance transport; however, hydration does not simply “dilute” the eutectic structure.^10^ Instead, a growing body of experimental and simulation work shows that water can reorganize local coordination environments, redistribute the stabilization balance between salt-HBD and ion–water contacts, and induce microheterogeneity or nanoscale segregation depending on composition and water content.^7,10–13^ These effects have been systematically discussed for Ionic Liquids (ILs)/DES-H_2_O mixtures, where nonlinear trends with hydration are commonly observed and where the microscopic origin of property changes is often traced to competitive solvation of the anion and the progressive restructuring of hydrogen-bond networks.^7,10–16^

From an application-oriented perspective, understanding the role of hydration in DESs is essential for their rational use across multiple technological fields. In separation processes, including extraction and purification, water can significantly modify solvent polarity, hydrogen-bonding ability, and solute partitioning, thereby directly affecting selectivity and efficiency.^2,17^ In electrochemical systems, such as batteries, supercapacitors, and electrodeposition media, hydration influences dielectric response, ionic mobility, and charge transport, which are central to electrolyte performance.^18,19^ Similarly, in biomass pretreatment and related materials-processing contexts, water is often used to reduce viscosity and improve mass transfer, while preserving key DES-enabled fractionation effects.^20,21^ Furthermore, a molecular-level understanding of hydration effects is necessary for establishing predictive design rules for DES formulation.

Within Type-III DESs, choline chloride (*ChCl*) is among the most widely used Hydrogen Bond Acceptor, and phenol constitutes a prototypical aromatic HBD capable of forming strong H-bond interactions with chloride. Phenol-based DESs have been experimentally characterized in terms of density, viscosity, and related thermophysical properties, and prior work has demonstrated that both temperature and HBD content significantly influence bulk behavior, consistent with the expectation that increasing phenol fraction modifies the connectivity and strength of the eutectic interaction network.^22^ While these experimental studies establish key trends, the molecular-level mechanisms governing how the eutectic organization evolves with HBD stoichiometry and controlled hydration remain insufficiently quantified; Particularly when multiple water contents are compared at fixed temperature and when the analysis is performed in a way that simultaneously links (i) interaction energetics, (ii) hydrogen-bond topology/dynamics, (iii) collective dielectric response, (iv) translational mobility, and (v) spatial organization captured by radial distribution functions.

Molecular dynamics (MD) simulations provide a direct route to connect these length scales, but reliability depends on validated interaction models for DES components and water. In this context, choline chloride-based DES force-field development and benchmarking have progressed substantially, enabling consistent reproduction of bulk properties and local structure across families of *ChCl* DESs.^23,24^ In parallel, multiple simulation and experimental studies on hydrated *ChCl* DESs (e.g., *ChCl*/urea and related systems) have shown that water preferentially coordinates chloride at low-to-moderate hydration and that higher water contents can promote clustering and more water-like connectivity, with direct consequences for structure and dynamics.^12,25^ These insights motivate extending systematic hydration analyses to *ChCl*-phenol DESs while explicitly resolving how the HBD ratio modulates the hydration response.

Here, we employ classical MD simulations to investigate deep eutectic solvents composed of choline chloride and phenol (*CCPhe*) under controlled variation of HBD stoichiometry and hydration. We consider three molar compositions (*ChCl*/phenol = 1:2, 1:3, and 1:4) and hydration levels from the anhydrous DES to 30% water added in 5% increments, allowing a consistent comparison of composition-dependent hydration effects. The central objective is to establish a coherent physicochemical picture of how water addition and HBD fraction reshape (i) the balance of stabilizing interactions within the eutectic network, (ii) the hydrogen-bond organization and dynamical persistence, (iii) collective polarization through dielectric observables and Kirkwood-type correlations, (iv) molecular mobility quantified via Einstein diffusion in the linear MSD regime, and (v) local structure described by RDFs referenced to choline. This multiproperty approach is essential because hydration effects in DESs are frequently nonmonotonic and strongly composition-dependent, as commonly observed in ionic liquid and related complex fluid mixtures, where competing interactions and structural reorganization lead to nonlinear behavior in transport, dielectric, and rheological properties.^26,27^

## 2 Computational Details

In this study, we employed molecular dynamics (MD) simulations to investigate the physicochemical properties of deep eutectic solvents (DESs) composed of choline chloride and phenol (*CCPhe*) under different hydrogen-bond donor (HBD) ratios. Systems with molar compositions of 1:2, 1:3, and 1:4 (*ChCl*/phenol) were examined. For each composition, various hydration levels were evaluated, starting from the pure solvent and gradually adding water in 5% increments up to 30%. Throughout this work, the systems are denoted as *CCPhe*_*x*_^*y*^, where *x* indicates the hydration level and *y* represents the molar ratio between choline chloride and the HBD. Table 1 provides detailed information on the composition of each simulated system. All initial configurations were generated using cubic simulation boxes with dimensions of 4 × 4 × 4 nm^3^. The final volumes obtained after equilibration and production runs are also reported in Table 1.

**Table 1:** Structural and Compositional Parameters of the Systems Investigated^a^,^b^

system	#CC	#HBD	#water molecules	#atoms	#water/#DES	volume box (nm^3^)	final density (kg/m^3^)
*CCPhe*_0%_^1:2^	175	350	0	8400	0.00	88.39	1076.9 ± 4.4
*CCPhe*_5%_^1:2^	165	330	160	8400	0.32	87.76	1077.9 ± 4.6
*CCPhe*_10%_^1:2^	158	316	135	7989	0.28	83.49	1078.2 ± 4.6
*CCPhe*_15%_^1:2^	149	298	179	7689	0.40	80.19	1077.7 ± 4.7
*CCPhe*_20%_^1:2^	140	280	261	7503	0.62	78.09	1075.7 ± 4.6
*CCPhe*_25%_^1:2^	131	262	293	7167	0.75	74.54	1073.8 ± 5.0
*CCPhe*_30%_^1:2^	122	244	389	7023	1.06	72.94	1069.5 ± 4.9
*CCPhe*_0%_^1:3^	145	435	0	8845	0.00	94.53	1074.8 ± 4.3
*CCPhe*_5%_^1:3^	131	393	89	8258	0.17	87.87	1074.1 ± 4.6
*CCPhe*_10%_^1:3^	136	408	117	8647	0.22	91.95	1073.9 ± 4.6
*CCPhe*_15%_^1:3^	122	366	183	7991	0.38	84.78	1071.9 ± 4.7
*CCPhe*_20%_^1:3^	116	348	209	7703	0.45	81.69	1070.9 ± 4.8
*CCPhe*_25%_^1:3^	109	327	238	7363	0.55	78.00	1069.3 ± 5.0
*CCPhe*_30%_^1:3^	102	306	302	7128	0.74	75.46	1066.2 ± 5.0
*CCPhe*_0%_^1:4^	115	460	0	8510	0.00	91.77	1072.4 ± 4.6
*CCPhe*_5%_^1:4^	108	432	178	8526	0.33	91.59	1067.9 ± 4.6
*CCPhe*_10%_^1:4^	104	416	123	8065	0.24	86.69	1069.6 ± 4.7
*CCPhe*_15%_^1:4^	98	392	176	7780	0.36	83.56	1067.6 ± 4.9
*CCPhe*_20%_^1:4^	92	368	234	7510	0.51	80.54	1064.4 ± 5.0
*CCPhe*_25%_^1:4^	86	344	314	7306	0.73	78.25	1060.9 ± 5.2
*CCPhe*_30%_^1:4^	80	240	622	6746	1.94	71.07	1050.5 ± 4.2

a This table reports the average simulation box volume (in nm^3^), the number of molecules of each component, the total number of atoms, and the water-to-DES (*CCPhe*) ratio for all simulated systems.

b CC = Choline and chloride pair; HBD = hydrogen-bond donor; DES = Deep eutectic solvents.

The number of molecules in each system was not fixed a priori, as the primary objective was to preserve the target molar ratios between choline chloride and phenol (1:2, 1:3, and 1:4) while systematically varying the hydration level. Therefore, the compositions were defined in terms of relative proportions rather than absolute molecule counts. As a consequence, the total number of choline chloride, phenol, and water molecules varies across systems to ensure that the intended stoichiometry is maintained for each condition. It is also important to consider that the molecular species involved differ significantly in size and interaction characteristics. To ensure physically consistent simulations, each system was constructed such that the simulation box is fully and homogeneously filled, avoiding artificial voids and ensuring stable densities under NPT conditions. This requires slight adjustments in the total number of molecules for each composition and hydration level. Additionally, small variations in the number of water molecules arise from the discrete nature of molecular counts combined with the requirement to simultaneously preserve molar ratios and maintain consistent system densities. These adjustments do not affect the overall composition trends but are necessary to ensure a robust and physically meaningful simulation setup.

All simulation boxes were initially constructed using Packmol software^28^ to generate a random distribution of molecules for the anhydrous DES systems. Hydrated systems were then obtained starting from these equilibrated pure configurations. For each target hydration level, a corresponding fraction of the DES components (choline chloride and phenol) was proportionally removed, and the resulting free volume was subsequently filled with water molecules using the *gmx solvate* tool available in the GROMACS package.^29^ It is important to note that the hydration level is therefore defined relative to the original DES composition. Due to the different molecular sizes and packing characteristics of choline chloride, phenol, and water, the number of water molecules inserted is not strictly proportional to the number of molecules removed. Consequently, small nonmonotonic variations in the number of water molecules may be observed between systems with different nominal hydration levels. These variations arise from geometric and packing constraints of the simulation box and do not affect the intended compositional trends.

Figure 01 illustrates the pure systems and those with the highest hydration level evaluated in this work (30%), along with the molecular structures of the components considered in the model. All systems were simulated using a multistage molecular dynamics (MD) protocol designed to ensure proper thermodynamic equilibration before the production stage. Initially, each model underwent a pre-equilibration phase of approximately 10 ns aimed at relaxing the system from the initial configuration and allowing the redistribution of kinetic and potential energy among the molecular degrees of freedom. During this stage, simulations alternated between the canonical (*NVT*) and isothermal–isobaric (*NPT*) ensembles in order to promote both thermal and volumetric equilibration of the system. In the *NVT* ensemble, the number of particles (*N*), system volume (*V*), and temperature (*T*) were maintained constant through the use of a thermostat, allowing the stabilization of the temperature and the establishment of an appropriate Maxwell–Boltzmann velocity distribution. This step primarily ensures proper thermalization of the system without modifying the simulation box dimensions. Subsequently, simulations were performed in the *NPT* ensemble, where the number of particles (*N*), pressure (*P*), and temperature (*T*) were kept constant through the combined use of thermostat and barostat algorithms. In this ensemble, the simulation box volume is allowed to fluctuate in response to the imposed pressure, enabling the system to relax its density and structural organization to values consistent with the target thermodynamic conditions (see Figure 02).

The alternation between *NVT* and *NPT* ensembles facilitates both thermal stabilization and density relaxation, which is particularly important for interfacial electrolyte systems where the initial configuration may not correspond to the equilibrium density or pressure. Thermodynamic equilibration was assessed by monitoring the time evolution of key macroscopic observables, including potential energy (see Supporting Information, Figures S1–S3). Once thermodynamic stability was achieved, each system proceeded to a 30 ns production simulation carried out in the *NPT* ensemble. Maintaining constant pressure and temperature during this stage ensures that the structural organization of the electrolyte and the electrode–electrolyte interface remains consistent with the equilibrium density of the system. During the production run, 30,000 trajectory frames were sampled at regular intervals and stored for subsequent analysis. These configurations were used to compute the structural and thermodynamic properties investigated in this work, including interfacial organization of ions, interaction energies, and other quantities relevant to the characterization of the electric double layer (EDL) at the graphene and graphyne electrode surfaces.

It is important to emphasize that the adequacy of the sampling was assessed through both dynamical and statistical convergence analyses. Diffusion coefficients were extracted from the linear regime of the mean square displacement (MSD), which was explicitly verified for all systems within the selected time window (see Supporting Information, Figures S4–S6), ensuring that the reported values correspond to the diffusive regime rather than short-time ballistic or subdiffusive behavior. In addition, the time evolution of the potential energy shows stable fluctuations around well-defined mean values with no systematic drift throughout the production runs. The corresponding energy distributions exhibit approximately Gaussian behavior across all compositions and hydration levels (Figures S1–S3), indicating consistent sampling of the configurational space. From a statistical standpoint, this Gaussian character is consistent with expectations from the central limit theorem for equilibrated systems, providing further evidence that the simulation length is sufficient to obtain reliable ensemble averages. Taken together, these results demonstrate that the production time employed in this work provides adequate convergence for the comparative and mechanistic analysis of hydration effects in *CCPhe* systems.

All simulations were carried out using a time step of 1 fs. Electrostatic interactions were computed using the Particle Mesh Ewald (PME) method,^30^ with a real-space cutoff of 1.2 nm and the potential-shift-Verlet scheme for short-range interactions. The same cutoff distance and modifier were applied to van der Waals (vdW) interactions. Long-range electrostatics were evaluated in reciprocal space using a PME mesh consistent with the GROMACS implementation.^29,30^ The system temperature was maintained at 298.15 K using the velocity-rescale (*v*-rescale) thermostat^31^ with a coupling constant of 0.1 ps, ensuring proper canonical ensemble sampling. For simulations performed in the *NPT* ensemble, the pressure was set to 1.013 bar and controlled using the Parrinello–Rahman barostat^32^ under isotropic coupling conditions, allowing uniform fluctuations of the simulation box volume.

Choline chloride and phenol molecules were described using the OPLS-DES force field parametrized by Acevedo et al.,^23^ which has been specifically developed to reproduce the structural and thermodynamic properties of deep eutectic solvents. Water molecules were modeled using the TIP3P model.^33^ All covalent bond lengths were constrained using the LINCS algorithm,^34^ enabling the use of the chosen integration time step while maintaining numerical stability. From the production trajectories, electrostatic and vdW interaction energies were extracted and analyzed. Hydrogen bonds (HBs) and their lifetimes were quantified using the geometric criteria and time correlation formalism proposed by Luzar and Chandler,^35,36^ with lifetime analysis performed according to the methodology implemented by van der Spoel and co-workers.^37^ HBs were identified using the geometric criteria implemented in the GROMACS analysis tools,^29^ adopting a donor–acceptor distance cutoff of 0.35 nm and a hydrogen–donor–acceptor angle cutoff of 30°. The same criteria were consistently applied to all interacting pairs to ensure a uniform comparison across different compositions and hydration levels. Molecular structures and trajectories were visualized using VMD,^38^ while all molecular dynamics simulations and trajectory analyses were performed using the GROMACS simulation package.^29^

It is important to note that the primary objective of this work is to provide a molecular-level understanding of hydration and composition effects in choline chloride–phenol DES systems, rather than to reproduce macroscopic thermophysical properties. The OPLS-DES force field employed here has been previously validated for choline chloride-based DESs, demonstrating reliable reproduction of structural organization and density in related systems. Therefore, the present study focuses on extracting mechanistic insights that are not directly accessible experimentally, such as interaction energy decomposition, hydrogen-bond dynamics, dipolar correlations, and detailed solvation structure under controlled hydration. While experimental data (e.g., density, viscosity, and conductivity) reported in the literature provide an important reference framework,^22^ the emphasis here is on elucidating the microscopic origin of these macroscopic behaviors.

## 3 Results and Discussion

### 3.1 Energetic Analysis

Pairwise interaction energies were obtained by summing the Coulombic and Lennard-Jones (LJ) contributions for each composition analyzed. For each molar ratio, only the *Cho*-*Cl*, *Cho*-*Phe*, *Cho*-Water, *Cl*-*Phe*, *Cl*-Water, and *Phe*-Water pairs were considered, and the total interaction energy was calculated as *E*_total_ = *E*_Coulomb_ + *E*_LJ_. The associated uncertainties were not estimated using a fixed relative error; instead, they were derived from the individual uncertainty values of each energetic contribution. The combined uncertainty of the total interaction energy was then obtained by uncertainty propagation model^39^ according to $u_{E_{total}}^{2}=u_{E_{Coulomb}}^{2}+u_{E_{LJ}}^{2}$ , where *u* represents each uncertainty, which is defined as the root-mean-square deviation (RMSD) per HBD. The plots were constructed as a function of hydration degree (0–30%) and fitted using linear regression, with the corresponding 95% confidence intervals and coefficients of determination (*R* ^2^) shown.

We emphasize that the interaction energies discussed in this work do not correspond to the total interaction energy of the system. Instead, they represent the short-range nonbonded interaction energy, described by the Coulomb short-range (Coul-SR) and Lennard-Jones short-range (LJ-SR) terms, as obtained from the standard GROMACS^29^ energy file (*.edr). In our simulations, a cutoff distance of 1.2 nm was adopted for both electrostatic and van der Waals interactions; therefore, these terms correspond to the real-space nonbonded interactions evaluated within this distance. The energy decomposition was performed using the GROMACS^29^ energy group scheme, which provides short-range nonbonded interaction energies between predefined molecular groups. Bonded terms (such as bonds, angles, and dihedrals), as well as electrostatic contributions from reciprocal space associated with the Particle Mesh Ewald (PME) method,^30^ were not included in this analysis. The purpose of this approach is not to reconstruct the full energetic decomposition of the solvated system, but rather to employ a consistent and physically meaningful metric to compare intermolecular interaction trends across different compositions and hydration levels. Accordingly, the reported interaction energies should be interpreted as ensemble-averaged, environment-dependent quantities that reflect relative changes in short-range intermolecular interactions.

In the anhydrous state, the 1:2 DES is energetically dominated by *Cho*-*Cl* and *Cl*-*Phe* interactions, with average values of approximately −43 and −36 kJ/mol per HBD, respectively. The *Cho*-*Phe* contribution is less intense (≈−22 kJ/mol per HBD), while *Phe*-*Phe* interactions are comparatively weak. This hierarchy indicates that the intrinsic cohesion of the eutectic mixture arises primarily from electrostatic stabilization between choline and chloride, complemented by strong chloride-phenol interactions. Upon progressive hydration up to 30%, a marked energetic redistribution is observed. The *Cl*-*Phe* interaction decreases from approximately −36 to −18 kJ/mol per HBD, corresponding to nearly a 50% reduction in stabilization. Similarly, *Cho*-*Cl* weakens from about −43 to −32 kJ/mol per HBD, representing a decrease of roughly 25%. *Cho*-*Phe* interactions follow the same trend, becoming progressively less stabilizing with increasing water content. In contrast, *Cl*-Water interactions emerge as the dominant stabilizing contribution at high hydration levels. While negligible in the pure system, this term reaches approximately −63 kJ/mol per HBD at 30% hydration, becoming the most energetically favorable interaction in the system. At this hydration level, *Cl*-Water is nearly three times more stabilizing than *Cl*-*Phe*, clearly indicating that the preferential coordination of chloride shifts from phenol to water. Additionally, *Phe*-*Phe* interactions become slightly more stabilizing (from approximately −1 to −7 kJ/mol per HBD), suggesting a reorganization of the phenolic environment as the original chloride-centered network weakens. Overall, hydration does not simply dilute the 1:2 DES; rather, it drives a substantial redistribution of the energetic balance. The intrinsic eutectic stabilization, initially governed by *Cho*-*Cl* and *Cl*-*Phe* interactions, is progressively replaced by chloride-water stabilization, fundamentally altering the internal cohesion of the system.

A similar qualitative behavior is observed for the 1:3 and 1:4 compositions, although the magnitude of the energetic redistribution depends strongly on the molar ratio. For the 1:3 system, the anhydrous state is characterized by strong *Cl*-*Phe* interactions (−33.85 kJ/mol per HBD) and moderate *Cho*-*Cl* (−23.28 kJ/mol per HBD) and *Cho*-*Phe* (−19.48 kJ/mol per HBD) contributions. Upon hydration, *Cl*-*Phe* becomes progressively less stabilizing, reaching −16.61 kJ/mol per HBD at 30% (Δ ≈ 17.24 kJ/mol, i.e., ∼51% weakening relative to the dry system). In parallel, chloride-water interactions emerge and intensify markedly: *Cl*-Water is already −13.15 kJ/mol per HBD at 5% hydration and reaches −39.75 kJ/mol per HBD at 30%, becoming the most stabilizing contribution at high hydration. The choline-centered interactions are comparatively less sensitive: *Cho*-*Cl* changes from −23.28 (0%) to −19.44 kJ/mol per HBD (30%) (Δ ≈ 3.84 kJ/mol, ∼17%), while *Cho*-*Phe* decreases from −19.48 to −13.70 kJ/mol per HBD (Δ ≈ 5.78 kJ/mol, ∼30%). Additionally, *Phe*-Water becomes increasingly stabilizing, evolving from −1.81 (5% hydrated system) to −6.11 kJ/mol per HBD (30% hydrated system), consistent with a growing contribution of water to the phenolic solvation environment.

In the 1:4 composition, the energetic reorganization induced by hydration is even more pronounced for the chloride-phenol interaction. In the anhydrous system, *Cl*-*Phe* is −30.37 kJ/mol per HBD, but it weakens dramatically to −6.39 kJ/mol per HBD at 30% hydration (Δ ≈ 23.98 kJ/mol, ∼79% weakening). Conversely, chloride-water stabilization becomes dominant at high hydration: *Cl*-Water increases from −19.42 kJ/mol per HBD at 5% hydrated system to −63.93 kJ/mol per HBD at 30% hydration level. In this composition, water also strengthens its interactions with both molecular components: *Cho*-Water decreases from −2.61 (5% hydrated system) to −14.56 kJ/mol per HBD (in 30% hydration level), and *Phe*-Water decreases from −3.92 (5%) to −10.08 kJ/mol per HBD (30%). Choline-chloride interactions remain comparatively unchanged (−14.77 at 0% vs −14.07 kJ/mol per HBD at 30%), while *Cho*-*Phe* weakens substantially (−17.01 to −8.82 kJ/mol per HBD; Δ ≈ 8.19 kJ/mol, ∼48%).

Taken together, these quantitative results establish a clear compositional hierarchy: 1:2 exhibits the strongest intrinsic eutectic stabilization and a large hydration-driven energetic inversion. 1:3 displays intermediate cohesive strength and a moderated redistribution, while 1:4 presents weaker initial cohesion and a dramatic suppression of *Cl*-*Phe* stabilization, although chloride-water interactions become equally strong at high hydration. Thus, although hydration systematically shifts stabilization toward chloride-water coordination in all *CCPhe* systems, the extent and structural consequences of this energetic rebalancing are strongly governed by the initial HBD ratio. The 1:2 composition combines high initial cohesion with substantial hydration-induced reorganization, whereas increasing phenol content progressively attenuates the robustness of the original eutectic network.

Still, it is worth pointing out that the larger uncertainties observed for the *Cho*-*Cl* interaction, particularly in systems with higher phenol content (1:4), can be attributed to the increased sensitivity of this pair to the local molecular environment. In these compositions, the presence of excess phenol and water leads to a highly competitive interaction landscape involving *Cho*-*Cl*, *Cho*-*Phe*, *Cl*-*Phe*, and solvent-mediated coordination. As a result, the direct ionic association between choline and chloride becomes less structurally constrained and exhibits more frequent fluctuations along the trajectory. This enhanced configurational variability leads to a broader distribution of interaction energies, and consequently to larger statistical uncertainties, reflecting the intrinsic heterogeneity of the system rather than a limitation of the methodology.

### 3.2 Hydrogen Bond Analysis

Hydrogen bonds were identified according to the standard Luzar-Chandler geometric and dynamical formalism.^35–37^ In this framework, a hydrogen bond between donor D and acceptor A exists when the intermolecular distance and angular criteria are simultaneously satisfied, typically defined by a donor–acceptor distance *r*_*DA*_ ≤ *r*_*c*_ and a hydrogen–donor–acceptor angle θ ≤ θ_*c*_. To characterize the dynamics of hydrogen bonding, Luzar and Chandler introduced the binary population operator *h*(*t*), defined as

$$h\left(t\right)=\{\begin{array}{l} 1,if\;a\;given\;pair\;is\;hydrogen-bonded\;at\;time\;t\;\\ 0,\;otherwise \end{array}$$ 1

The time autocorrelation function of hydrogen-bond existence is then written as

2 $$C\left(t\right)=\frac{\left\langle h\left(0\right)h\left(t\right)\right\rangle }{\left\langle h\right\rangle }$$

where ⟨*h*⟩ is the equilibrium probability of hydrogen-bond formation. The average lifetime τ is obtained from the time integral of the correlation function

3 $$\tau =\int_{0}^{\infty }C\left(t\right)dt$$

which provides a measure of the persistence of hydrogen-bonded configurations. In this work, both the average number of hydrogen bonds and their lifetimes were normalized per hydrogen-bond donor (HBD) to allow a consistent comparison among systems with different compositions and hydration levels. The results are shown in Table 2 as follows. The hydrogen-bond populations are reported per hydrogen-bond donor (HBD) to provide an intensive, composition-independent metric that enables direct comparison across systems with different stoichiometries; although the resulting absolute values are numerically small, they reflect the probability of bond formation per donor site, such that even modest variations correspond to significant relative changes and clearly capture the structural reorganization induced by hydration.

**Table 2:** Average Number of Hydrogen Bonds Per HBD and Corresponding Average Hydrogen-Bond Lifetimes Per HBD (ps) for *CCPhe* Systems at Molar Ratios 1:2, 1:3, and 1:4 as a Function of Hydration Level (0–30%)

average number of HBs per HBD																		
hydration level	*CCPhe* ^1:2^						*CCPhe* ^1:3^						*CCPhe* ^1:4^					
	Cho-Cho	Cho-Phe	Cho-H_2_O	Phe-Phe	Phe-H_2_O	H_2_O-H_2_O	Cho-Cho	Cho-Phe	Cho-H_2_O	Phe-Phe	Phe-H_2_O	H_2_O-H_2_O	Cho-Cho	Cho-Phe	Cho-H_2_O	Phe-Phe	Phe-H_2_O	H_2_O-H_2_O
Pure	0.02	0.05	-	0.04	-	-	0.01	0.05	-	0.07	-	-	0.00	0,05	-	0,11	-	-
5%	0.01	0.06	0.07	0.06	0.16	0.15	0.01	0.05	0.03	0.09	0.12	0.05	0.00	0.05	0.05	0.15	0.22	0.19
10%	0.01	0.05	0.07	0.07	0.13	0.12	0.01	0.05	0.04	0.10	0.14	0.08	0.00	0.05	0.04	0.14	0.17	0.11
15%	0.01	0.05	0.10	0.08	0.17	0.24	0.01	0.05	0.06	0.11	0.22	0.22	0.00	0.05	0.05	0.14	0.24	0.22
20%	0.01	0.06	0.13	0.09	0.26	0.51	0.01	0.05	0.08	0.13	0.23	0.32	0.00	0.04	0.07	0.17	0.28	0.41
25%	0.01	0.05	0.15	0.11	0.28	0.70	0.01	0.05	0.09	0.13	0.28	0.43	0.00	0.04	0.09	0.20	0.30	0.75
30%	0.01	0.05	0.19	0.13	0.35	1.26	0.01	0.04	0.11	0.16	0.30	0.74	0.01	0.03	0.20	0.22	0.40	2.99

average HBs-lifetime per HBD (ps)																		
hydration level	*CCPhe* ^1:2^						*CCPhe* ^1:3^						*CCPhe* ^1:4^					
	Cho-Cho	Cho-Phe	Cho-H_2_O	Phe-Phe	Phe-H_2_O	H_2_O-H_2_O	Cho-Cho	Cho-Phe	Cho-H_2_O	Phe-Phe	Phe-H_2_O	H_2_O-H_2_O	Cho-Cho	Cho-Phe	Cho-H_2_O	Phe-Phe	Phe-H_2_O	H_2_O-H_2_O
Pure	0.23	0.17	-	0.14	-	-	0.09	0.07	-	0.08	-	-	0.02	0.01	-	0.02	-	-
5%	0.10	0.09	0.18	0.10	0.41	0.76	0.06	0.05	0.10	0.07	0.26	0.44	0.01	0.01	0.01	0.01	0.04	0.08
10%	0.10	0.10	0.22	0.10	0.56	0.89	0.06	0.05	0.12	0.07	0.26	0.56	0.01	0.01	0.02	0.01	0.05	0.11
15%	0.10	0.08	0.20	0.10	0.40	0.72	0.05	0.04	0.07	0.06	0.20	0.29	0.01	0.01	0.01	0.01	0.04	0.07
20%	0.07	0.07	0.11	0.08	0.27	0.41	0.01	0.01	0.02	0.02	0.05	0.11	0.01	0.01	0.01	0.01	0.03	0.06
25%	0.06	0.05	0.10	0.07	0.25	0.33	0.01	0.01	0.02	0.02	0.05	0.09	0.01	0.01	0.01	0.01	0.02	0.04
30%	0.04	0.04	0.05	0.06	0.17	0.20	0.01	0.01	0.01	0.01	0.04	0.06	0.01	0.01	0.01	0.02	0.02	0.03

In the anhydrous state, the three *CCPhe* compositions display distinct hydrogen-bond networks. For the 1:2 system, *Cho*-*Phe* and *Phe*-*Phe* interactions dominate the hydrogen-bond population (for anhydrous state), whereas hydration progressively shifts the network toward water-mediated hydrogen bonds (see Table 2), with 0.05 and 0.04 bonds per HBD, respectively, while *Cho*-*Cho* contributes 0.02. Increasing the phenol fraction increase phenolic connectivity: in 1:3, *Phe*-*Phe* increases to 0.07 per HBD, and in 1:4 it reaches 0.11 per HBD. Thus, even in the absence of water, increasing the HBD ratio promotes a phenol-centered hydrogen-bond network. Hydration introduces a restructuring of this network. In the 1:2 system, at 30% water content, *Cho*-Water and *Phe*-Water reach 0.19 and 0.35 hydrogen bonds per HBD, respectively, while Water-Water rises to 1.26 per HBD, becoming the most abundant hydrogen-bond contribution. Simultaneously, *Phe*-*Phe* increases from 0.04 to 0.13 per HBD, whereas *Cho*-*Cho* decreases from 0.02 to 0.01. This indicates that water does not merely replace intrinsic DES hydrogen bonds but also promotes additional phenolic self-association alongside extensive water clustering. For the 1:3 composition at 30% hydration, *Cho*-Water and *Phe*-Water reach 0.11 and 0.30 per HBD, respectively, and Water-Water attains 0.74 per HBD, substantially lower than the 1.26 observed in 1:2. *Phe*-*Phe* increases to 0.16 per HBD, slightly exceeding the value found in 1:2. These results show that increasing phenol content moderates water clustering while maintaining a significant participation of phenol in the hydrogen-bond network, predominantly through phenol-water interactions, leading to a more balanced hydrogen-bond structure.

The dynamical analysis reveals that hydration reduces hydrogen-bond persistence. In the anhydrous 1:2 system, the lifetimes per HBD are 0.23 ps for *Cho*-*Cho*, 0.17 ps for *Cho*-*Phe*, and 0.14 ps for *Phe*-*Phe*. In 1:3, these decrease to 0.09, 0.07, and 0.08 ps, and in 1:4 they drop further to approximately 0.02–0.01 ps, demonstrating progressively higher intrinsic mobility as the HBD fraction increases. At 30% hydration, hydrogen bond’s lifetimes decrease markedly for all compositions. In 1:2, *Cho*-*Cho* and *Cho*-*Phe* both fall to 0.04 ps, *Phe*-*Phe* to 0.06 ps, while *Phe*-Water and Water-Water reach 0.17 and 0.20 ps, respectively. For 1:3, most hydrogen bond’s lifetimes lie between 0.01 and 0.04 ps, and Water-Water is approximately 0.06 ps. In 1:4, nearly all hydrogen bond’s lifetimes approach 0.01–0.02 ps, with Water-Water around 0.03 ps. Thus, although water increases the average number of hydrogen bonds, these interactions are comparatively short-lived, particularly in compositions with higher phenol content.

The population and dynamical analyses of the hydrogen bonds reveal two simultaneous hydration effects: (1st) an increase in water-mediated hydrogen bonds and (2^sd^) a decrease in hydrogen-bond persistence. The overall structural robustness follows the hierarchy 1:2 > 1:3 > 1:4, with 1:2 retaining the most persistent network under hydration and 1:4 exhibiting the most dynamic and water-clustered regime, demonstrating that increasing HBD fraction enhances baseline mobility and amplifies the dynamical character of the hydrated DES systems. In contrast, the 1:4 system exhibits the most differentiated water structuring. At 30% hydration, Water-Water reaches ∼3 hydrogen bonds per HBD, more than double the value observed in 1:2 and four times that of 1:3. *Phe*-Water increases to 0.40 per HBD, and *Phe*-*Phe* reaches 0.22 per HBD, the highest among all compositions. This indicates extensive water aggregation coexisting with a dense phenolic framework, reflecting a highly heterogeneous hydrogen-bond landscape. Moreover, a particularly pronounced increase in the Water-Water hydrogen-bond population is observed for the 1:4 system at 30% hydration. This behavior is associated with the strong structural reorganization induced at the highest water content in this phenol-rich composition. Because the hydrogen-bond populations are normalized per HBD, the substantially larger water/DES ratio in the 30% system amplifies the reported Water-Water contribution. Physically, this indicates that at high hydration the 1:4 mixture evolves toward a more heterogeneous regime with enhanced water aggregation, in which Water-Water connectivity becomes significantly more prominent than in the other compositions.

### 3.3 Dielectric Behavior, Kirkwood Correlations, and Diffusive Dynamics

To connect the energetic redistribution and hydrogen-bond reorganization discussed previously with macroscopic collective properties, we evaluated the static dielectric constant, the infinite-system Kirkwood factor, and the translational diffusion coefficient for all *CCPhe* compositions. The static dielectric constant was obtained from fluctuations of the total dipole moment of the simulation cell according to ref:^40^

4 $$\varepsilon =1+\frac{4\pi }{3Vk_{B}T}\left(\left\langle M^{2}\right\rangle -{\left\langle M\right\rangle }^{2}\right),$$

where *V* is the system volume, *k*_*B*_ the Boltzmann constant, *T* the temperature, and *M* the total dipole moment. This formulation directly links macroscopic dielectric response to microscopic dipolar fluctuations, making it particularly sensitive to the orientational organization induced by hydration. The orientational correlations between dipoles were quantified using the Kirkwood factor

5 $$g_{K}=\frac{\left\langle M^{2}\right\rangle }{N\mu^{2}},$$

which measures the degree of collective alignment among molecular dipoles. Values of *g*_*K*_ > 1 indicate preferential parallel correlations, whereas *g*_*K*_ ≈ 1 corresponds to nearly random orientation. Because *g*_*K*_ reflects long-range dipolar correlations, it is interpreted in the thermodynamic (infinite-system) limit. Finite simulation boxes under periodic boundary conditions may artificially constrain long-range correlations and distort collective polarization effects. Therefore, adopting the infinite-system framework ensures that the reported *g*_*K*_ values represent bulk orientational correlations and are consistent with macroscopic dielectric theory.

Translational mobility was quantified using the Einstein relation^40^

6 $$D=\lim_{t\to \infty }\frac{1}{6t}\left\langle {\left|\mathrm{r⃗}\left(t\right)-\mathrm{r⃗}\left(0\right)\right|}^{2}\right\rangle ,$$

where diffusion coefficients were extracted from the linear region of the mean square displacement between 0 and 10 ns. This time window corresponds to the diffusive regime, in which MSD increases linearly with time, excluding the initial ballistic and transient subdiffusive regimes typical of strongly hydrogen-bonded liquids. Restricting the analysis to this interval ensures that the reported diffusion coefficients reflect true long-time transport behavior. All MSD graphs in this time region are available in the Supporting Information (see Figures S4–S6), while the static dielectric constant, the infinite-system Kirkwood factor, and the translational diffusion coefficient are shown in Table 03. It is important to highlight that the Kirkwood factor is interpreted in the infinite-system limit and reflects collective dipolar correlations derived from total dipole moment fluctuations; therefore, values close to unity indicate weak long-range orientational cooperativity, even in systems with strong local electrostatic structuring.

**Table 03:** Static Dielectric Constant (ε), Infinite-System Kirkwood Factor (*g*_*K*_), and Diffusion Coefficient (*D*, × 10^–5^ cm^2^ s^–1^) for *CCPhe* Systems at Molar Ratios 1:2, 1:3, and 1:4 under Hydration Levels from 0 to 30%

system	ε	*g*_*K*_	*D* (×10^–5^cm^2^/s)
*CCPhe*_0%_^1:2^	4.74	1.00	0.0096 ± 0.0005
*CCPhe*_5%_^1:2^	6.84	1.16	0.0241 ± 0.0013
*CCPhe*_10%_^1:2^	6.01	1.03	0.0206 ± 0.0022
*CCPhe*_15%_^1:2^	7.30	1.18	0.0269 ± 0.0002
*CCPhe*_20%_^1:2^	6.89	0.98	0.0500 ± 0.0029
*CCPhe*_25%_^1:2^	7.85	1.06	0.0672 ± 0.0019
*CCPhe*_30%_^1:2^	9.84	1.19	0.1025 ± 0.0174
*CCPhe*_0%_^1:3^	5.71	1.36	0.0183 ± 0.0025
*CCPhe*_5%_^1:3^	5.77	1.16	0.0380 ± 0.0001
*CCPhe*_10%_^1:3^	5.48	1.05	0.0479 ± 0.0018
*CCPhe*_15%_^1:3^	6.07	1.04	0.0635 ± 0.0031
*CCPhe*_20%_^1:3^	6.59	1.09	0.0769 ± 0.0084
*CCPhe*_25%_^1:3^	6.73	1.05	0.0966 ± 0.0007
*CCPhe*_30%_^1:3^	8.23	1.17	0.1368 ± 0.0141
*CCPhe*_0%_^1:4^	4.48	1.10	0.0497 ± 0.0025
*CCPhe*_5%_^1:4^	5.89	1.09	0.1193 ± 0.0068
*CCPhe*_10%_^1:4^	5.27	1.04	0.0871 ± 0.0019
*CCPhe*_15%_^1:4^	6.35	1.16	0.1083 ± 0.0036
*CCPhe*_20%_^1:4^	7.34	1.22	0.1553 ± 0.0015
*CCPhe*_25%_^1:4^	8.69	1.28	0.1889 ± 0.0057
*CCPhe*_30%_^1:4^	15.93	1.55	0.3930 ± 0.0191

In the anhydrous systems, the dielectric constant already reflects compositional differences in collective dipolar fluctuations. The 1:3 composition exhibits the highest dielectric constant (ε = 5.71), followed by 1:2 (ε = 4.74) and 1:4 (ε = 4.48). This ordering is consistent with the hydrogen-bond analysis, where 1:3 displayed the strongest intrinsic phenolic connectivity in the absence of water. The Kirkwood factor follows the same trend in the dry systems: *g*_*K*_ = 1.36 for 1:3, compared to 1.10 for 1:4 and 1.00 for 1:2 composition, indicating more pronounced intrinsic orientational correlations in the 1:3 composition. In contrast, the diffusion coefficient (reported in units of 10^–5^ cm^2^/s) increases with HBD fraction in the dry state: *D* = 0.0096 for 1:2, 0.0183 for 1:3, and 0.0497 for 1:4. Thus, while 1:3 exhibits the strongest dipolar correlations in the absence of water, 1:4 composition is intrinsically the most mobile system, consistent with its shorter hydrogen-bond lifetimes and weaker electrostatic cohesion discussed previously. Hydration induces a clear amplification of dielectric response in all systems, although the magnitude and correlation behavior differ. In the 1:2 system, ε increases from 4.74 to 9.84 at 30% hydration, corresponding to more than a 2-fold enhancement. The Kirkwood factor rises from 1.00 to 1.19, indicating moderate growth in cooperative dipolar alignment. Simultaneously, the diffusion coefficient increases from 0.0096 to 0.1025 (10^–5^ cm^2^/s), representing an order-of-magnitude increase in molecular mobility. This behavior is consistent with the energetic inversion toward chloride-water interactions and the reduction in hydrogen-bond lifetimes, which collectively enhance dipolar fluctuations while preserving some structural organization.

For the 1:3 system, ε increases from 5.71 to 8.23 at 30% hydration, a smaller relative amplification compared to 1:2 system. The Kirkwood factor decreases from 1.36 in the pure system to 1.17 at 30% hydration, indicating that hydration weakens the strong intrinsic orientational correlations originally present in this composition. Despite this reduction in *g*_*K*_, the diffusion coefficient increases steadily from 0.0183 to 0.1368 (10^–5^ cm^2^/s), reflecting enhanced mobility driven by hydrogen-bond weakening and progressive water incorporation. Therefore, in 1:3, hydration reduces intrinsic dipolar cooperativity while still increasing overall dielectric response through enhanced dipole fluctuations. The 1:4 system exhibits the most pronounced collective response at high hydration. The dielectric constant increases from 4.48 to 15.93 at 30% hydration, representing the largest absolute and relative enhancement among all compositions. The Kirkwood factor rises substantially from 1.10 to 1.55, indicating strong cooperative dipolar correlations under high water content. The diffusion coefficient reaches 0.393 (10^–5^ cm^2^/s), nearly eight times the dry value, confirming the emergence of a highly dynamic regime. This behavior is fully consistent with the extensive water clustering (Water-Water ∼3 HBs per HBD) and the strong energetic shift toward chloride-water stabilization previously identified.

Taken together, the results reveal a composition-dependent interplay between dipolar correlations and molecular mobility. In the dry systems, 1:3 exhibits the strongest orientational correlations, whereas 1:4 is intrinsically the most mobile. At high hydration (30%), the hierarchy in dielectric amplification and mobility becomes 1:4 > 1:2 > 1:3 for ε and *D*, while cooperative dipolar correlations follow 1:4 > 1:2 ≈ 1:3. Thus, increasing HBD fraction enhances the susceptibility of the system to hydration-induced polarization and transport amplification. These macroscopic observables provide a collective manifestation of the microscopic energetic redistribution and hydrogen-bond reorganization described in the previous subsections, completing the multiscale picture of hydration-driven structural and dynamical evolution in *CCPhe* systems (see Figure 04).

**Figure 04:**
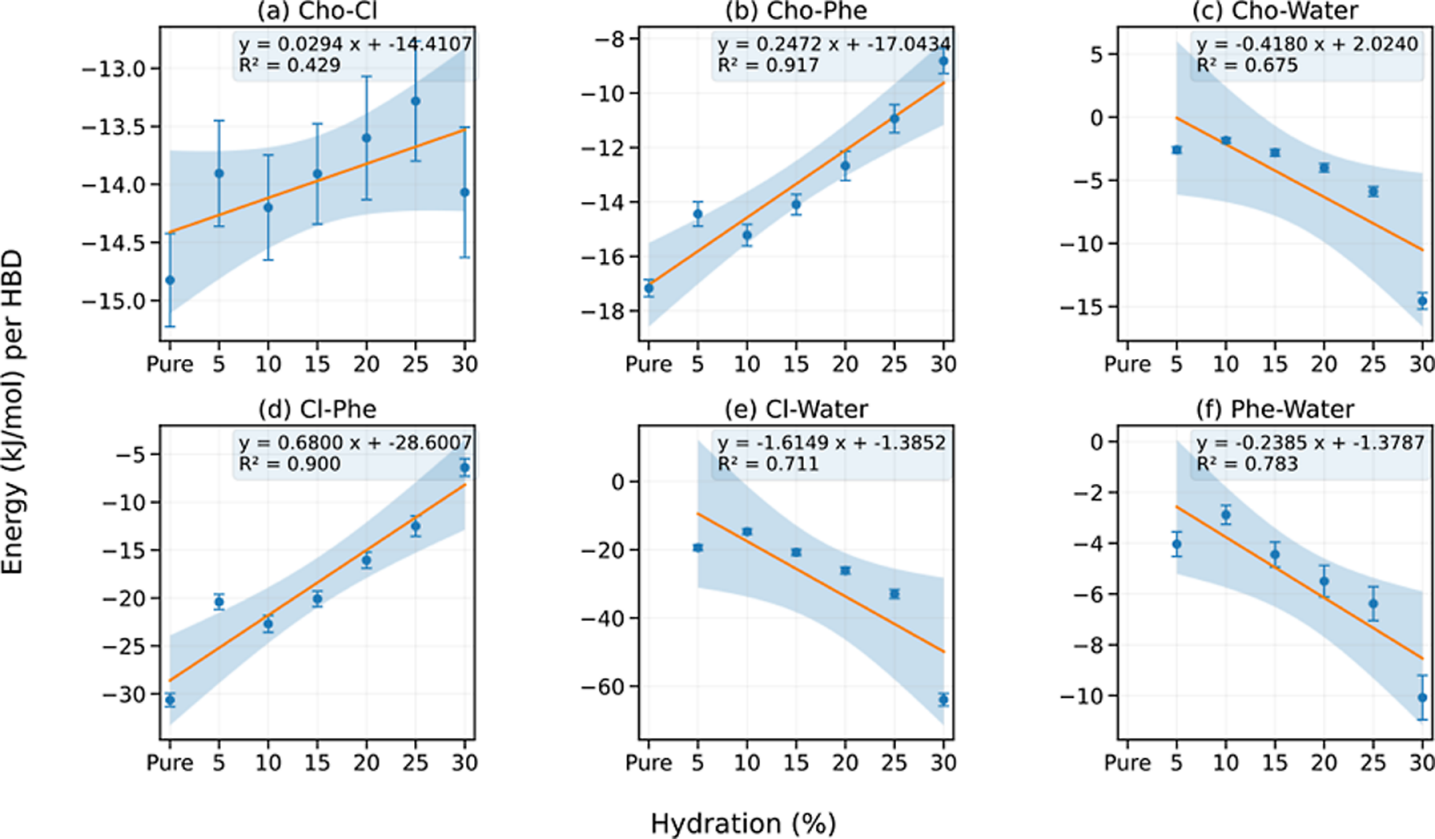
Average short-range interaction energy (Coulomb + Lennard-Jones) per HBD (kJ mol^–1^ per HBD) as a function of hydration level for the (a) *Cho*-*Cl*, (b) *Cho*-*Phe*, (c) *Cho*-Water, (d) *Cl*-*Phe*, (e) *Cl*-Water, and (f) *Phe*-Water pairs in the *CCPhe* ^1:4^ system. Symbols correspond to average values from the production trajectories and error bars. Linear regressions (*R* ^2^ values shown in each panel) illustrate the pronounced energetic reorganization induced by hydration.

It is important to note that the relationship between density (Table 1) and dielectric constant in the *CCPhe* systems reveals opposite trends as the hydration level increases. As shown in the correlation plots, the introduction of water systematically increases the dielectric constant while slightly reducing the density of the systems. Quantitatively, for the 1:2 composition, the dielectric constant increases from 4.74 to 9.84 when the hydration level rises from 0 to 30%, while the density decreases from 1076.9 to 1069.5 kg/m^3^, corresponding to a reduction of approximately 7.4 kg/m^3^. A similar behavior is observed for the 1:3 systems, where ε increases from 5.71 to 8.23, while the density decreases from 1074.8 to 1066.2 kg/m^3^ (Δρ ≈ 8.6 kg/m^3^). The effect becomes even more pronounced for the 1:4 composition, in which ε increases dramatically from 4.48 to 15.93, accompanied by a more significant density decrease from 1072.4 to 1050.5 kg/m^3^ (Δρ ≈ 21.9 kg/m^3^). The negative correlation between these two properties is clearly observed in the linear fits shown in Figure 05, with correlation coefficients of *R* ^2^ = 0.665, 0.932, and 0.971 for the 1:2, 1:3, and 1:4 systems, respectively.

**Figure 05:**
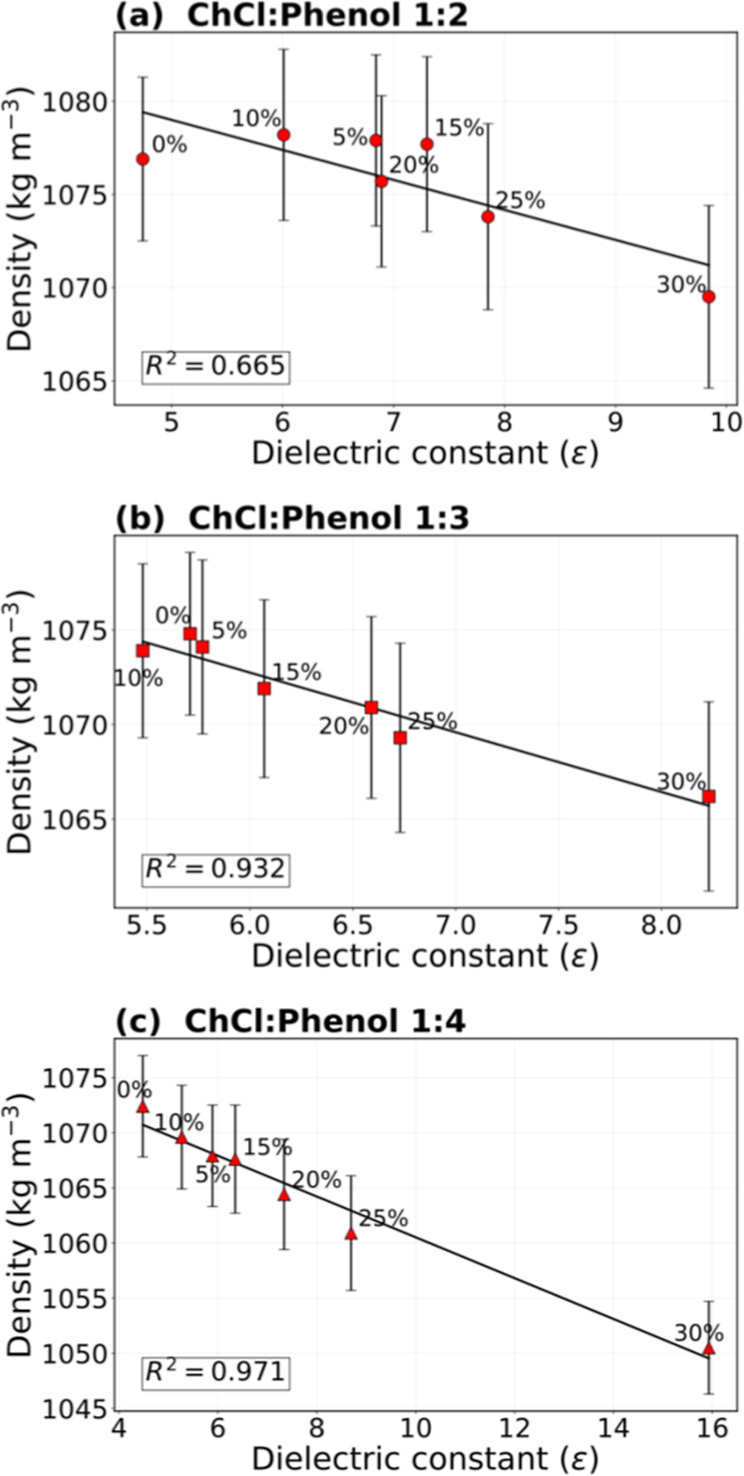
Relationship between density (ρ) and dielectric constant (ε) for the hydrated CCPhe systems at different ChCl/phenol molar ratios. Panels (a–c) correspond to the compositions 1:2, 1:3, and 1:4, respectively. Each point represents a system with a specific hydration level (0–30%), indicated next to the markers, while the error bars correspond to the uncertainty in the calculated density values. The black lines represent linear fits highlighting the correlation between the two properties.

These values indicate that the inverse relationship between density and dielectric constant becomes progressively stronger as the phenol content increases. From a molecular perspective, this behavior arises from the dual role of water in the DES structure. On one hand, water disrupts and reorganizes the hydrogen-bond network characteristic of the deep eutectic solvent, slightly expanding the liquid structure and therefore reducing the overall density. On the other hand, water molecules possess a high dipole moment, which enhances the collective dipolar fluctuations of the system and significantly increases its dielectric response. Consequently, although the systems become slightly less densely packed upon hydration, their ability to respond to an external electric field increases markedly due to the enhanced polarity and orientational freedom of the molecular dipoles. This effect is particularly evident for the 1:4 systems, where the stronger correlation between ε and density highlights the dominant role of dipolar polarization over structural packing in determining the dielectric properties of these hydrated DES mixtures.

### 3.4 Radial Distribution Function

To further elucidate the microscopic structural rearrangements induced by hydration, radial distribution functions (RDFs) were calculated for Choline (*Cho*)-centered correlations with chloride, phenol, and water. The RDF *g*_*ij*_(*r*) describes the probability of finding a particle *j* at a distance *r* from a reference particle *i*, relative to an ideal gas at the same density, and is defined as

7 $$g_{ij}\left(r\right)=\frac{1}{4\pi r^{2}\rho_{j}}\left\langle \sum_{k\in j}\delta \left(r-r_{ik}\right)\right\rangle$$

The structural organization of the first solvation shell can be quantified by the height and position of the first peak, while its integrated area up to the first minimum provides the coordination number. Because choline was used as the reference in all cases, the RDFs directly reflect how the local environment around the choline cation evolves with composition and hydration. The results for 1:2, 1:3 and 1:4 system are highlighted in Figures 06 and 08, respectively. Additional RDFs for chloride–phenol, chloride–water, water–water, and phenol–phenol pairs were also calculated and are provided in the Supporting Information (Figures S7–S15) (see Figure 07).

**Figure 06:**
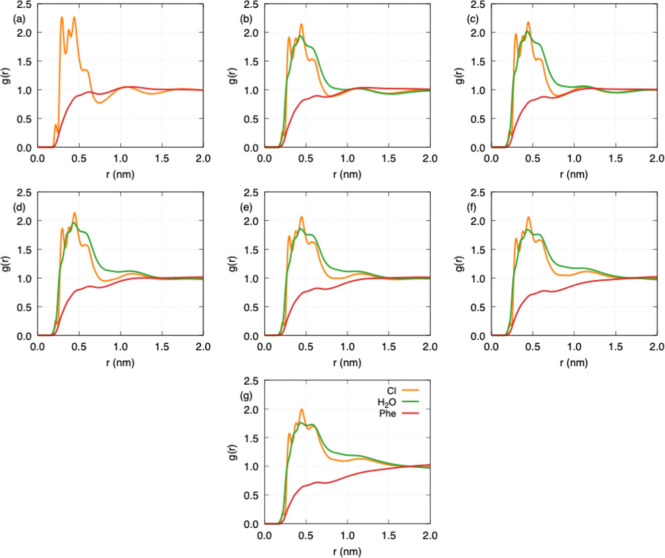
Radial distribution functions *g*(*r*) between choline (reference) and *Cl* ^–^, Water, and phenol for the *CCPhe* ^1:2^ system at different hydration levels as follows: (a) 0%, (b) 5%, (c) 10%, (d) 15%, (e) 20%, (f) 25%, and (g) 30%.

**Figure 07:**
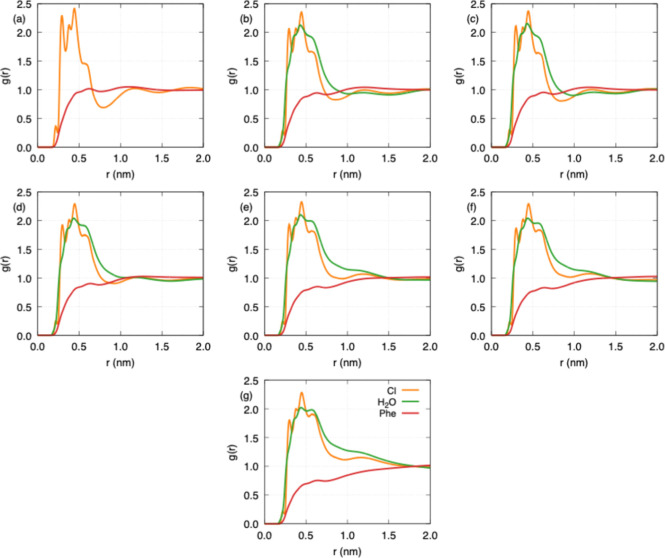
Radial distribution functions *g*(*r*) calculated using choline as the central reference and considering correlations with chloride (orange), water (green), and phenol (red) in the *CCPhe* ^1:3^ system. Panels correspond to (a) 0%, (b) 5%, (c) 10%, (d) 15%, (e) 20%, (f) 25%, and (g) 30% hydration.

**Figure 08:**
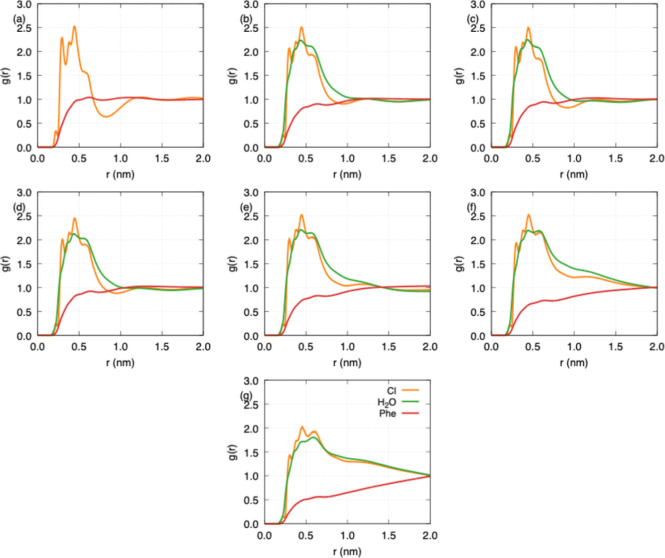
Radial distribution functions *g*(*r*) describing *Cho*-*Cl* (orange), *Cho*-Water (green), and *Cho*-*Phe* (red) correlations in the *CCPhe* ^1:4^ system under different hydration conditions: (a) pure, (b) 5%, (c) 10%, (d) 15%, (e) 20%, (f) 25%, and (g) 30% of water.

In the anhydrous systems, the *Cho*-*Cl* correlation exhibits the most pronounced first peak among all pairs. For 1:2, the first *Cho*-*Cl* peak is located near 0.45–0.50 nm with a maximum intensity close to 2.3, indicating strong electrostatic association between choline and chloride. In 1:3, the peak remains near the same position but slightly increases in intensity (≈2.4), while in 1:4 it reaches approximately 2.5, suggesting that although energetic cohesion is weaker in 1:4, local choline-chloride contact probability remains high. In contrast, *Cho*-*Phe* correlations show much lower first peaks, *g*(*r*) ≈ 0.9–1.0, indicating weaker direct structural organization between choline and phenol in the dry DES. Upon hydration, significant structural reorganization is observed. In 1:2, increasing water content progressively reduces the intensity of the *Cho*-*Cl* first peak, particularly at 30% hydration, where it decreases to approximately 2.0. Simultaneously, a pronounced *Cho*-Water peak emerges at roughly 0.45–0.50 nm, reaching intensities comparable to or slightly below the *Cho*-*Cl* peak. This structural inversion is consistent with the energetic shift toward chloride-water stabilization identified earlier and with the increased population of water-mediated hydrogen bonds. The *Cho*-*Phe* correlation remains comparatively weak and broad, indicating that hydration preferentially reorganizes the ionic domain rather than enhancing choline-phenol structuring.

The 1:3 system exhibits a more moderate redistribution. Although the *Cho*-*Cl* peak decreases with hydration, its intensity remains relatively high even at 30% of Water. The *Cho*-Water peak grows steadily but does not surpass the *Cho*-*Cl* peak as clearly as in 1:2. This behavior is consistent with the smaller energetic inversion and the partial reduction of intrinsic dipolar correlations observed in the Kirkwood factor analysis. Structurally, 1:3 retains a more balanced ionic-hydrogen-bond network even under hydration. The most pronounced structural transformation occurs in 1:4. While the dry system already shows strong *Cho*-*Cl* association, hydration leads to a substantial increase in *Cho*-Water correlation intensity, particularly at 25–30% of Water, where the *Cho*-Water peak approaches or exceeds 2.2–2.3. Concurrently, the *Cho*-*Cl* peak becomes less dominant relative to water coordination. This is consistent with the dramatic increase in dielectric constant (ε = 15.93 at 30% hydration) and the large Kirkwood factor (1.55), indicating enhanced collective polarization driven by water-rich local environments. The broadening of the second solvation region in 1:4 further suggests increased structural heterogeneity, in agreement with the high diffusion coefficient (0.393 × 10^–5^ cm^2^/s). Thus, the RDF results provide direct structural evidence of the hydration-induced redistribution previously identified at the energetic and hydrogen-bond levels. Hydration progressively replaces choline-chloride coordination with water-rich local environments, modifies dipolar correlations, and facilitates enhanced molecular mobility. The magnitude of this reorganization follows the same compositional dependence observed in dielectric and transport properties, completing the structural–energetic–dynamic framework of *CCPhe* systems.

Taken together, the results obtained across the different analyses reveal a coherent multiscale picture of hydration effects in *CCPhe* systems. At the molecular level, hydration promotes a progressive energetic redistribution, in which chloride-phenol and choline-chloride interactions are weakened and replaced by increasingly favorable chloride-water coordination. This shift directly impacts the hydrogen-bond network, leading to an increase in water-mediated hydrogen bonds accompanied by a systematic reduction in hydrogen-bond lifetimes, indicating enhanced dynamical flexibility. These microscopic changes propagate to collective properties: the increase in dipolar fluctuations and partial reorganization of orientational correlations are reflected in the growth of the dielectric constant and the evolution of the Kirkwood factor. Simultaneously, the weakening and shortening of hydrogen-bond interactions reduce structural constraints, resulting in significantly enhanced molecular mobility, as evidenced by the diffusion coefficients. From a structural perspective, these effects are captured by the RDF analysis, which shows that hydration does not disrupt the local organization but instead induces a competitive restructuring of the first solvation shell around choline, with water progressively replacing chloride as a coordinating species. Thus, these results demonstrate that hydration acts as a unifying control parameter that links interaction energetics, hydrogen-bond topology, collective polarization, and transport properties. The observed composition-dependent response further highlights that the balance between structural robustness and dynamic adaptability can be systematically tuned through both HBD ratio and water content, providing a consistent physicochemical framework for understanding and designing hydrated DES systems.

To provide a quantitative description of the structural reorganization induced by hydration, coordination numbers (CNs) were calculated by integrating the choline-centered RDFs up to the first minimum as follows:

8 $$N\left(r\right)=4\pi \rho \int_{0}^{r_{\min}}g\left(r\right)r^{2}dr$$

These results are summarized in Table 04. In the anhydrous systems, the first solvation shell of choline is primarily composed of phenol molecules, with CN values of approximately 7.28, 8.80, and 9.88 for the 1:2, 1:3, and 1:4 compositions, respectively. In contrast, chloride coordination is significantly lower, ranging from 3.39 to 4.00. This indicates that, despite the strong electrostatic interactions between choline and chloride, the local environment around choline is structurally dominated by phenol molecules, particularly at higher HBD ratios.

**Table 04:** Coordination Numbers (CN) Derived from the Integration of Choline-Centered Radial Distribution Functions (RDFs) up to the First Minimum for *CCPhe* Systems at Molar Ratios 1:2, 1:3, and 1:4 as a Function of Hydration (0–30%)^a^

system	species	CN	system	species	CN	system	species	CN
CCPhe_0%_^1:2^	Cl	4.00	CCPhe_0%_^1:3^	Cl	3.66	CCPhe_0%_^1:4^	Cl	3.39
	Phe	7.28		Phe	8.80		Phe	9.88
CCPhe_5%_^1:2^	Cl	5.26	CCPhe_5%_^1:3^	Cl	4.89	CCPhe_5%_^1:4^	Cl	4.96
	Water	7.15		Water	4.39		Water	19.07
	Phe	5.95		Phe	7.91		Phe	7.98
CCPhe_10%_^1:2^	Cl	5.17	CCPhe_10%_^1:3^	Cl	4.64	CCPhe_10%_^1:4^	Cl	4.58
	Water	6.22		Water	5.45		Water	6.95
	Phe	6.35		Phe	8.08		Phe	8.46
CCPhe_15%_^1:2^	Cl	6.09	CCPhe_15%_^1:3^	Cl	5.40	CCPhe_15%_^1:4^	Cl	4.95
	Water	8.79		Water	23.63		Water	22.76
	Phe	6.06		Phe	7.11		Phe	7.82
CCPhe_20%_^1:2^	Cl	6.42	CCPhe_20%_^1:3^	Cl	5.55	CCPhe_20%_^1:4^	Cl	5.46
	Water	37.29		Water	29.47		Water	36.99
	Phe	5.37		Phe	6.68		Phe	6.88
CCPhe_25%_^1:2^	Cl	6.45	CCPhe_25%_^1:3^	Cl	5.86	CCPhe_25%_^1:4^	Cl	5.89
	Water	43.12		Water	39.41		Water	56.25
	Phe	5.18		Phe	5.92		Phe	5.65
CCPhe_30%_^1:2^	Cl	6.65	CCPhe_30%_^1:3^	Cl	5.93	CCPhe_30%_^1:4^	Cl	14.58
	Water	62.04		Water	47.51		Water	112.33
	Phe	4.32		Phe	5.91		Phe	3.45

a The CN values quantify the average number of *Cl*, water, and phenol molecules in the first solvation shell of choline.

Upon hydration, a clear reorganization of the first solvation shell is observed. The coordination of water increases dramatically with increasing hydration level for all compositions. For example, in the 1:2 system, the *Cho*-Water coordination number increases from zero in the anhydrous system to approximately 62.0 at 30% hydration. Similar trends are observed for the 1:3 and 1:4 systems, where *Cho*-Water reaches ∼47.5 and ∼112.3, respectively, indicating a strong accumulation of water in the vicinity of choline at high hydration levels.

Simultaneously, the phenol coordination decreases with increasing water content. In the 1:2 system, *Cho*-*Phe* decreases from 7.28 in the pure DES to 4.32 at 30% hydration, while in the 1:4 system it decreases more sharply from 9.88 to 3.45. This reduction reflects the progressive displacement of phenol molecules from the first solvation shell as water becomes the dominant coordinating species. The behavior of chloride coordination is more complex. In the 1:2 and 1:3 systems, *Cho*-*Cl* increases moderately with hydration, reaching values around 6.6 and 5.9 at 30% hydration, respectively. In contrast, the 1:4 system exhibits a pronounced increase, with *Cho*-*Cl* reaching ∼14.6 at 30% hydration. This suggests that, in addition to direct coordination, chloride ions may participate in extended or water-mediated structures around choline at high hydration levels, particularly in phenol-rich systems. Thus, the coordination number analysis provides clear quantitative evidence that hydration induces a substantial restructuring of the local environment around choline. The first solvation shell evolves from a phenol-dominated arrangement in the anhydrous DES to a water-rich environment at high hydration levels, with chloride ions remaining structurally relevant through both direct and indirect coordination. These findings strongly support the competitive coordination mechanism inferred from the RDF analysis and are fully consistent with the energetic and hydrogen-bond trends discussed in previous sections.

## 4 Conclusions

In this study, we performed a comprehensive molecular dynamics investigation of choline chloride-phenol deep eutectic solvents (*CCPhe*) at molar ratios of 1:2, 1:3, and 1:4 under systematically increasing hydration levels. By combining energetic, hydrogen-bond, dielectric, transport, and structural analyses, we established a consistent multiscale picture of how water incorporation modifies the physicochemical behavior of these DES systems. At the energetic level, hydration promotes a progressive redistribution of stabilization from intrinsic chloride–phenol and choline–chloride interactions toward chloride–water interactions. This redistribution is composition dependent, with the 1:2 system exhibiting stronger initial eutectic cohesion, while higher HBD fractions facilitate a more pronounced water-driven energetic reorganization. These energetic changes are directly reflected in the hydrogen-bond network, where increasing water content enhances water-mediated interactions while simultaneously reducing the lifetimes of the original DES hydrogen bonds, particularly at high hydration levels. The dielectric constant and infinite Kirkwood factor further demonstrate how these microscopic rearrangements translate into collective dipolar behavior, with hydration systematically amplifying dipolar fluctuations and cooperative orientational correlations. The most pronounced dielectric enhancement occurs for the 1:4 composition at elevated water contents, indicating stronger collective polarization as the hydrogen-bond network becomes increasingly reorganized.

This increase in collective polarization is accompanied by substantial growth in translational mobility, as evidenced by the diffusion coefficients obtained from the long-time linear regime of the mean square displacement. The diffusion coefficients, expressed in units of × 10^–5^ cm^2^/s, increase by nearly an order of magnitude between dry and highly hydrated systems, confirming that water incorporation promotes a significant dynamic softening of the DES network. Radial distribution function analysis provides direct structural evidence of this reorganization. Hydration modifies the composition of the first solvation shell around choline by introducing competitive coordination between chloride and water while preserving the characteristic contact distance. Rather than inducing complete structural disruption, water progressively reshapes the local solvation environment through replacement and competition mechanisms, leading to mixed ionic–aqueous coordination at high hydration levels. These structural changes underpin the observed dielectric amplification and mobility enhancement. Overall, our results demonstrate that hydration acts as a powerful tuning parameter in *CCPhe* systems, simultaneously influencing energetic balance, hydrogen-bond connectivity, dipolar correlations, and molecular transport. The interplay between composition and hydration determines the extent of structural flexibility and collective polarization, with higher phenol fractions exhibiting greater susceptibility to hydration-induced reorganization at elevated water contents. This integrated multiscale analysis provides fundamental insight into the molecular mechanisms governing hydrated deep eutectic solvents and establishes a framework for rationally modulating their physicochemical properties through controlled water incorporation.

## Supplementary Material



## Notes

Potential energy convergence analysis for all compositions and hydration levels (Figures S1–S3); mean square displacement (MSD) profiles employed in the determination of diffusion coefficients (Figures S4–S6); additional radial distribution functions (RDFs) for *Cl*-Water, *Cl*-phenol, Water–Water, and phenol–phenol correlations across all systems and hydration conditions (Figures S7–S15), providing complementary structural characterization (PDF)
