# Supplementary material for: Hydration and Molar Ratio Effects in Choline Chloride-Phenol Deep Eutectic Solvents
Source: J Phys Chem B. 2026 May 20;130(23):5923–38. doi: 10.1021/acs.jpcb.6c01636 (PMC13267076; doi:10.1021/acs.jpcb.6c01636)
Supplement: Supplementary file 1 [file jp6c01636_si_001.pdf]

S U P P O R T   I N F O R M A T I O N

Hydration and Molar Ratio Effects in Choline Chloride-Phenol Deep Eutectic Solvents

Lucas de S. Silva[1] and Guilherme Colherinhas[1]*

[1] *Instituto de Física, Universidade Federal de Goiás, 74690-900, Goiânia, GO, Brazil.*

* *gcolherinhas@ufg.br*

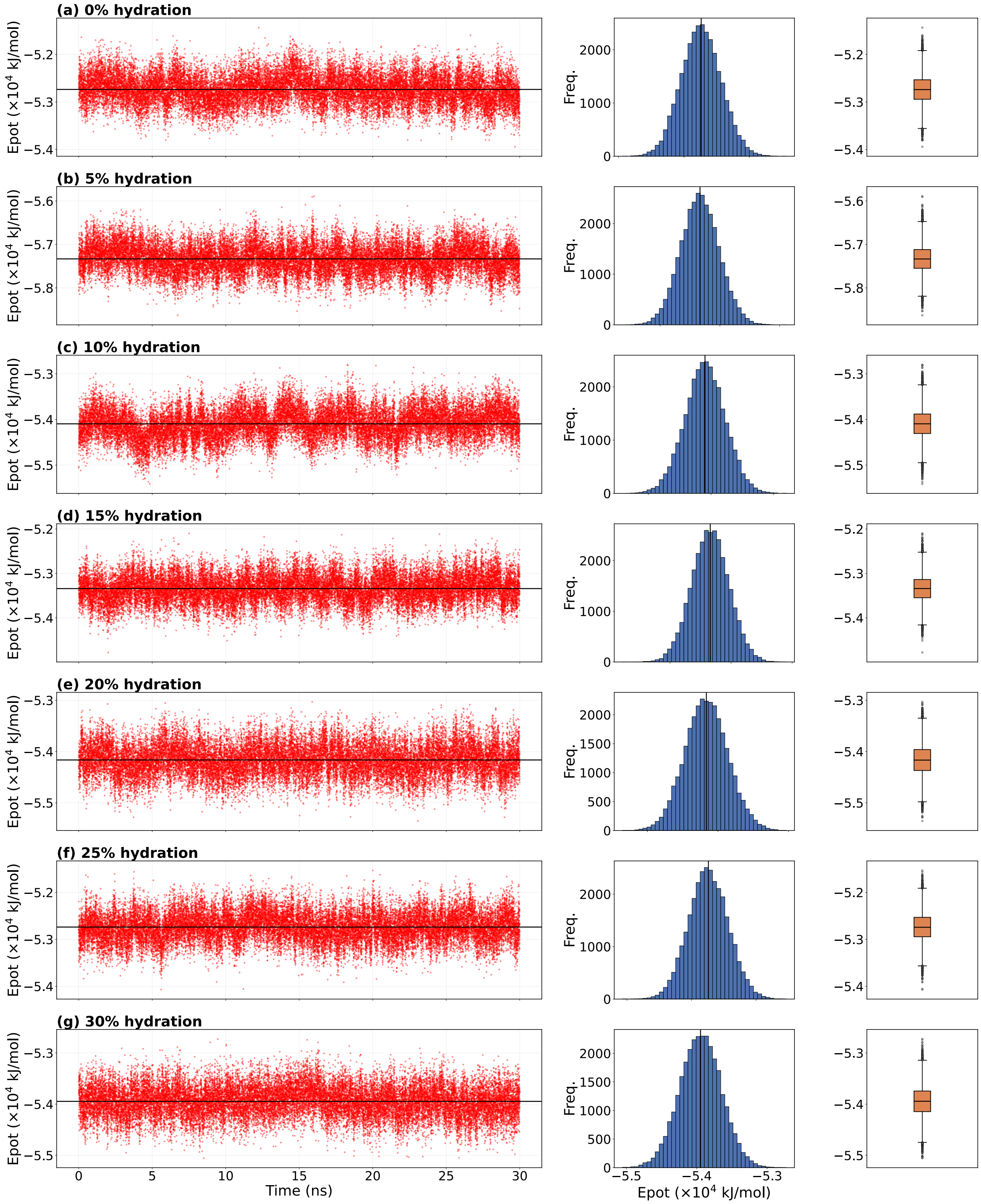

Figure S1 - Convergence analysis of the potential energy for the systems with a 1:2 Choline chloride–phenol molar ratio at different hydration levels (0–30%). Panels (a–g) correspond to increasing water contents from 0% to 30%. For each system, the potential energy as a function of simulation time is shown as a scatter plot (left), illustrating the temporal stability of the trajectory. The middle panels present the corresponding histograms, revealing the statistical distribution of the potential energy values (approximately Gaussian distributions). The boxplots on the right summarize the dispersion, median, and presence of possible outliers.

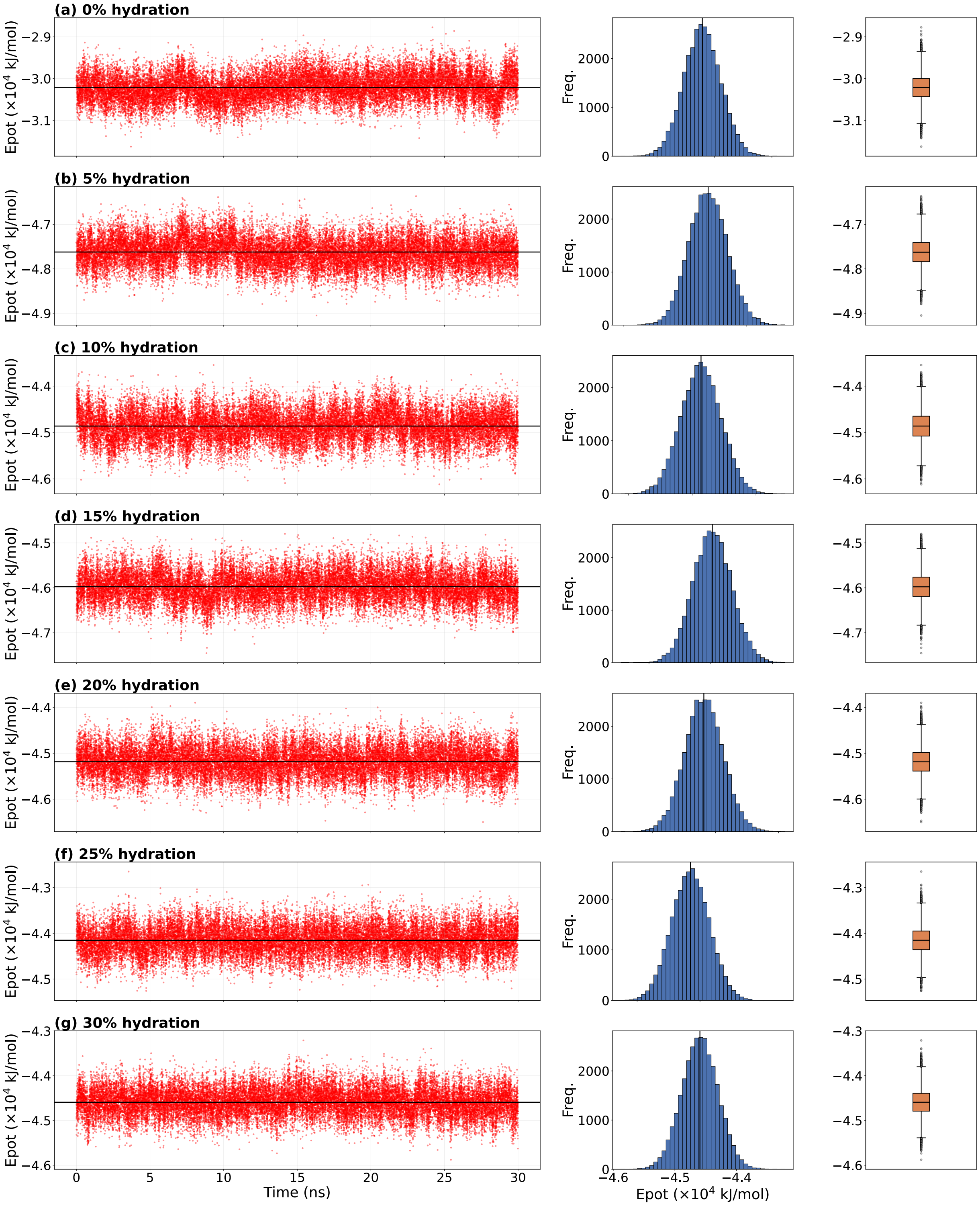

Figure S2 - Convergence analysis of the potential energy for the systems with a 1:3 Choline chloride–phenol molar ratio under different hydration levels (0–30%). Panels (a–g) represent the systems with increasing water content. The left column displays the evolution of the potential energy along the simulation time, allowing the assessment of energetic stability. The central panels show the histograms of the potential energy values, illustrating the statistical distribution sampled during the trajectory. The boxplots on the right provide a compact representation of the spread and central tendency of the energy distributions. The absence of systematic drifts and the symmetric distributions confirm that the simulations achieved equilibrium.

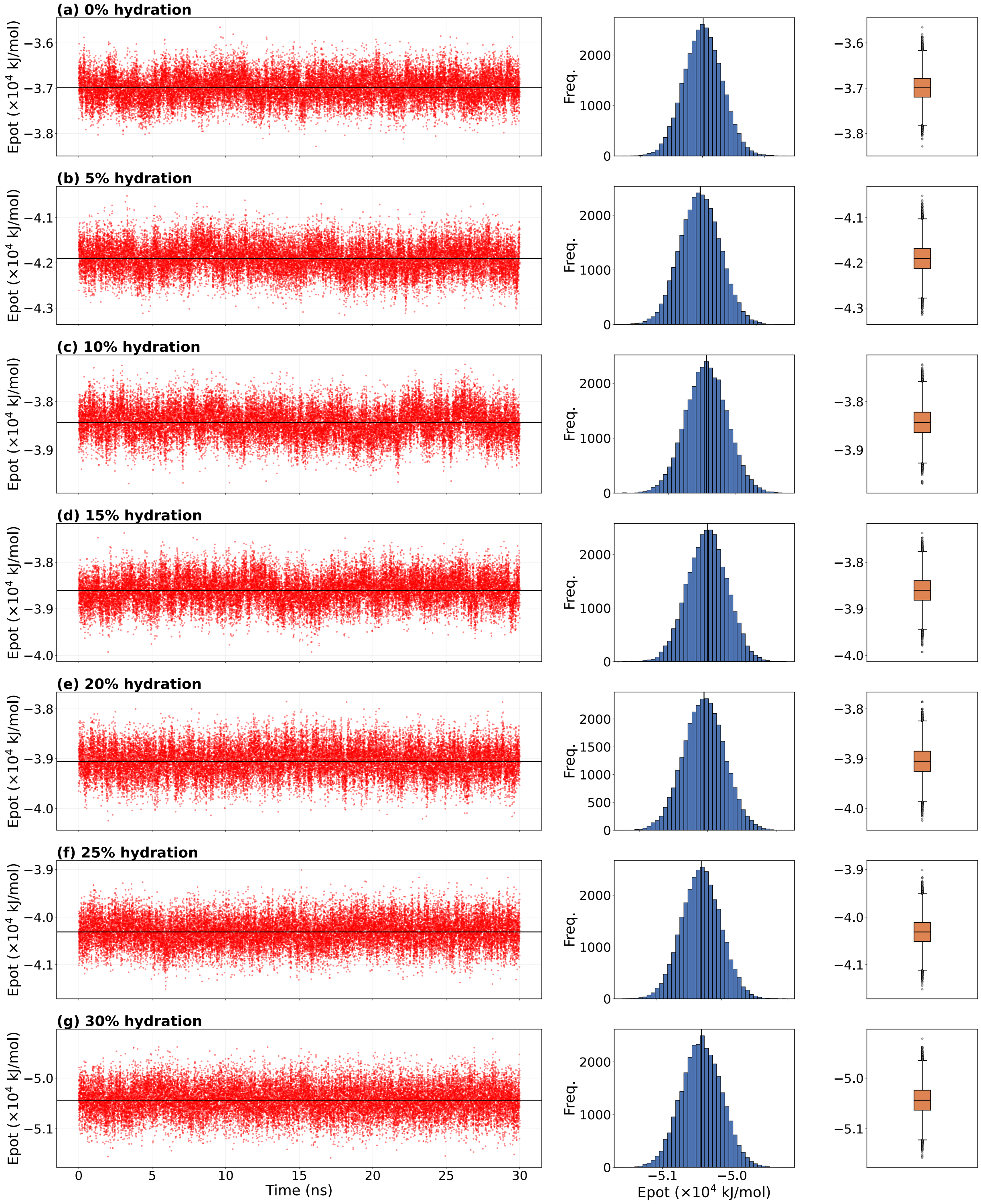

Figure S3 - Convergence analysis of the potential energy for the systems with a 1:4 Choline chloride–phenol molar ratio at hydration levels ranging from 0% to 30%. Panels (a–g) correspond to increasing water content. For each hydration level, the left plots show the temporal fluctuations of the potential energy throughout the molecular dynamic's trajectory. The middle plots present the corresponding energy histograms, while the boxplots on the right summarize the distribution characteristics, including median, interquartile range, and outliers. The relatively stable fluctuations and near-Gaussian distributions indicate that the systems are well equilibrated.

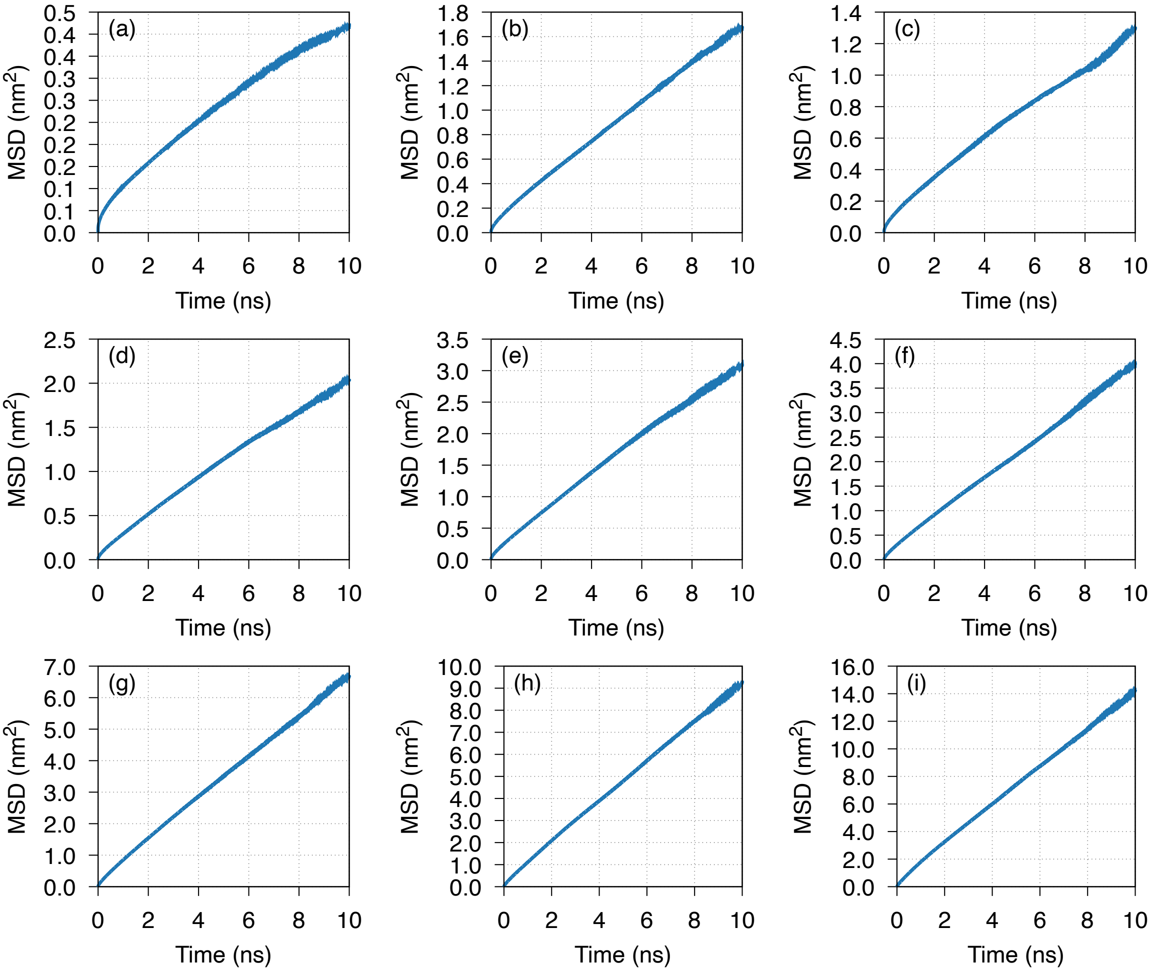

Figure S4 - Mean square displacement (MSD) as a function of time for the $CCPhe^{1:2}$ system at increasing hydration levels: (a) pure DES, (b) 5%, (c) 10%, (d) 15%, (e) 20%, (f) 25%, and (g) 30% H_2O.

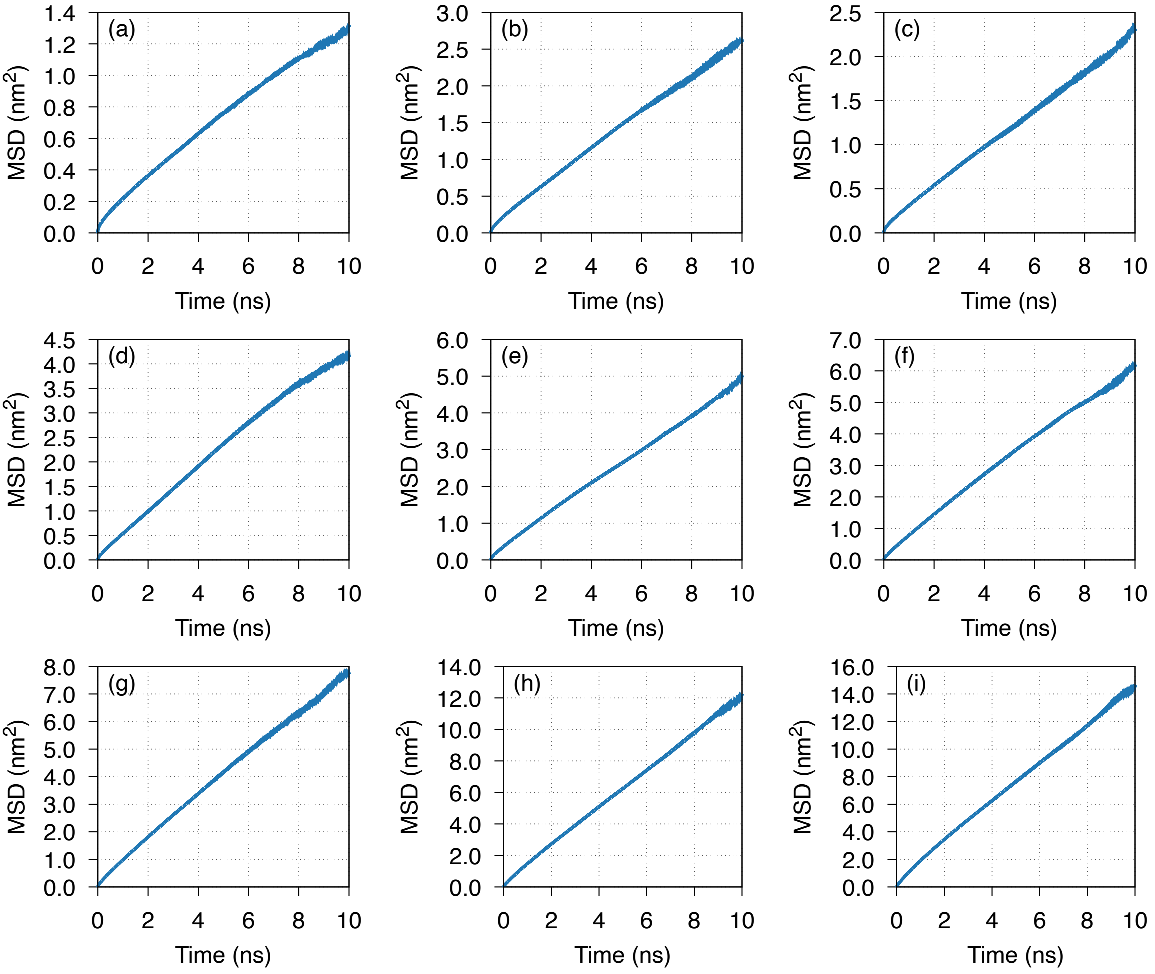

Figure S5 - Time-dependent mean square displacement (MSD) curves for the $CCPhe^{1:3}$ composition under different hydration conditions: (a) 0%, (b) 5%, (c) 10%, (d) 15%, (e) 20%, (f) 25%, and (g) 30% water content.

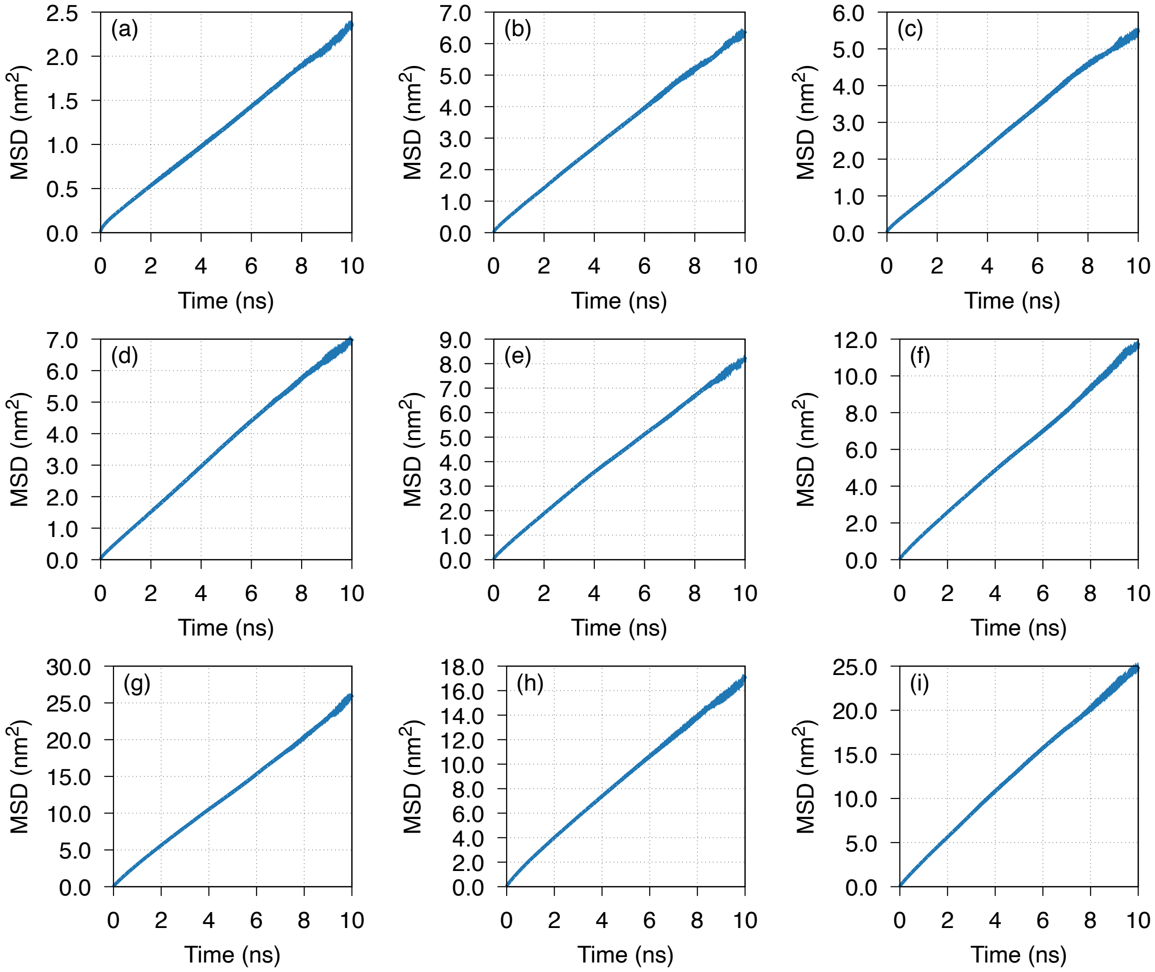

Figure S6 - Mean square displacement (MSD) profiles for the $CCPhe^{1:4}$ system at progressively increasing hydration: (a) pure DES, (b) 5%, (c) 10%, (d) 15%, (e) 20%, (f) 25%, and (g) 30% H_2O.

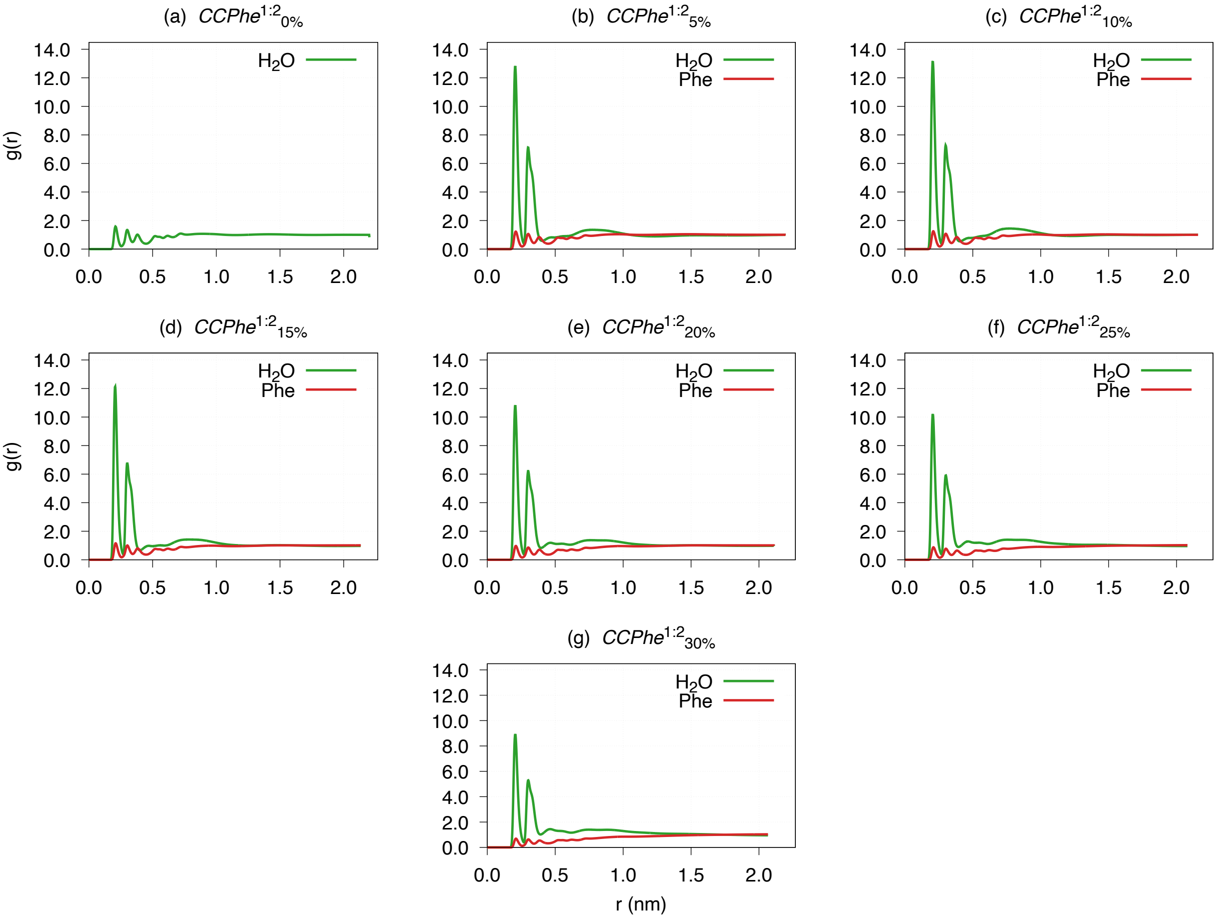

Figure S7 - Radial distribution functions $g(r)$ describing $Cl^{-} - H_2O$ (green) and $Cl^{-} - Phe$ (red) correlations in the $CCPhe^{1:2}$ system under different hydration conditions: (a) pure, (b) 5%, (c) 10%, (d) 15%, (e) 20%, (f) 25%, and (g) 30% H_2O.

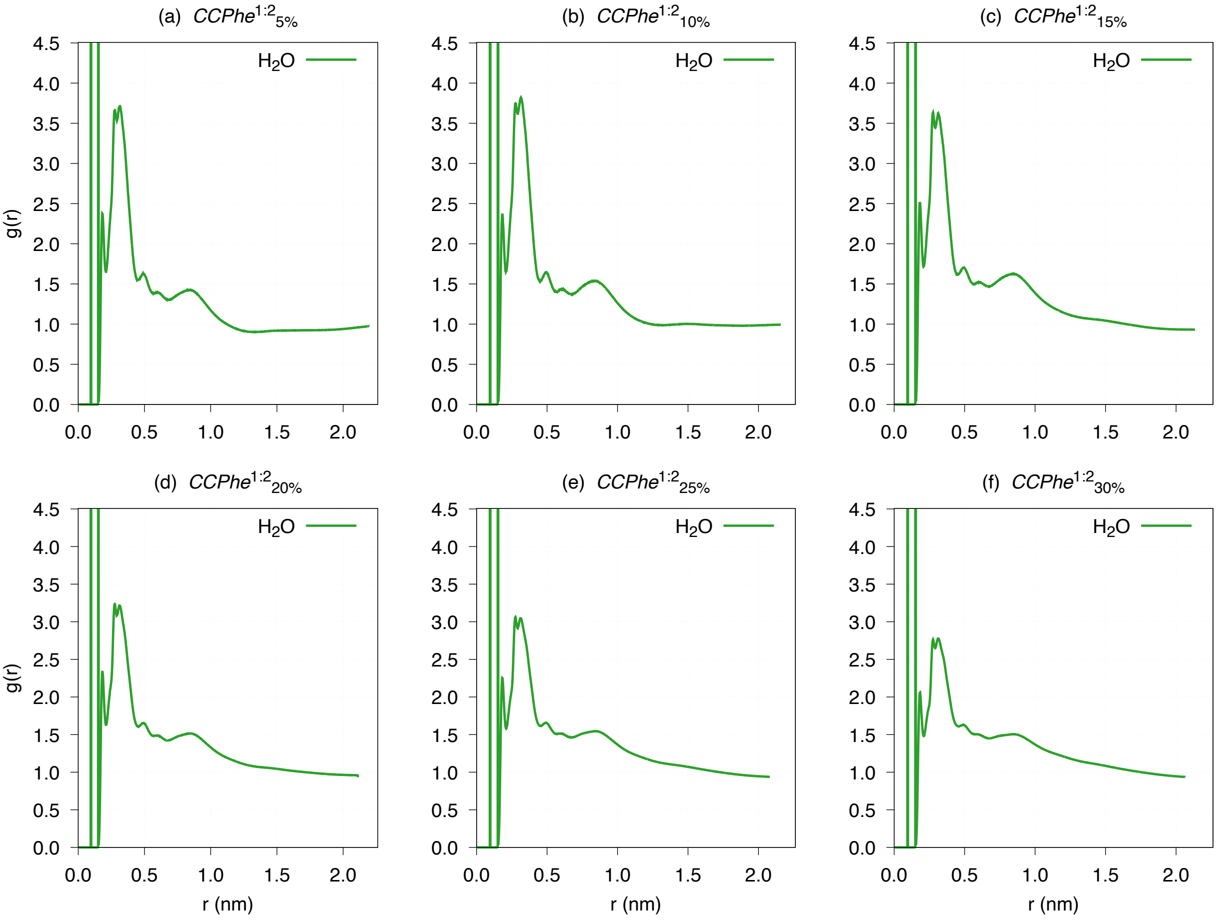

Figure S8 - Radial distribution functions $g(r)$ describing $H_2O - H_2O$ (green) correlations in the $CCPhe^{1:2}$ system under different hydration conditions: (a) 5%, (b) 10%, (c) 15%, (d) 20%, (e) 25%, and (f) 30% H_2O.

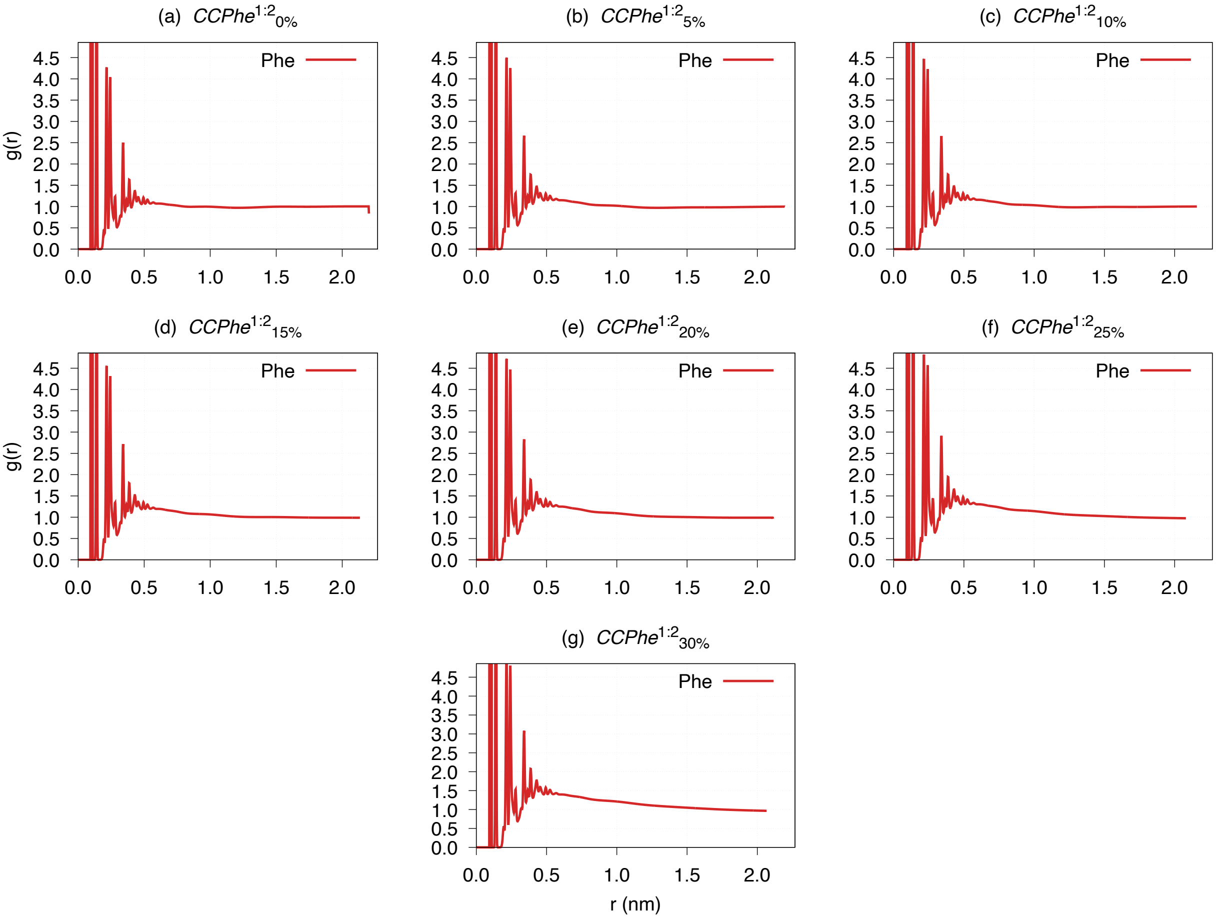

Figure S9 - Radial distribution functions $g(r)$ describing $Phe - Phe$ (red) correlations in the $CCPhe^{1:2}$ system under different hydration conditions: (a) pure, (b) 5%, (c) 10%, (d) 15%, (e) 20%, (f) 25%, and (g) 30% H_2O.

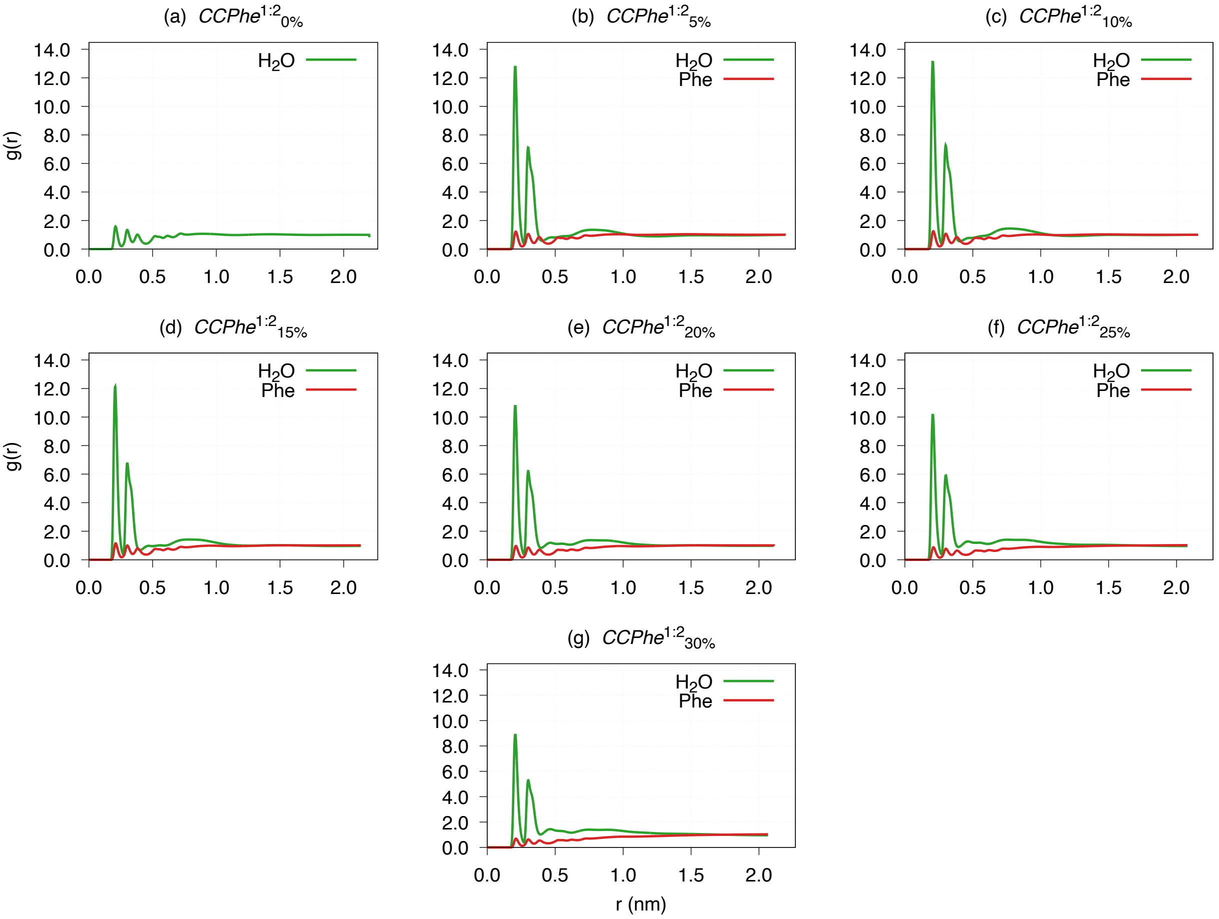

Figure S10 - Radial distribution functions $g(r)$ describing $Cl^{-} - H_2O$ (green) and $Cl^{-} - Phe$ (red) correlations in the $CCPhe^{1:3}$ system under different hydration conditions: (a) pure, (b) 5%, (c) 10%, (d) 15%, (e) 20%, (f) 25%, and (g) 30% H_2O.

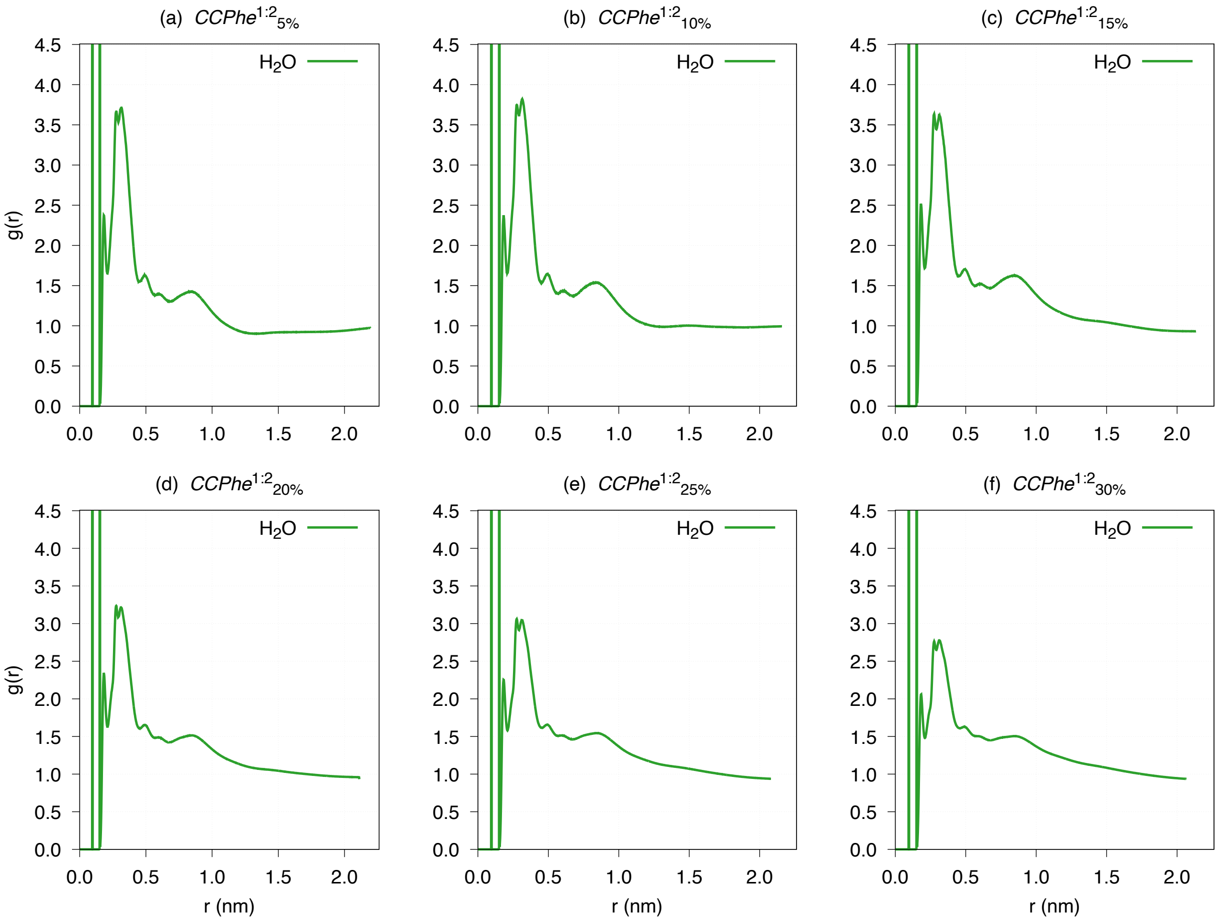

Figure S11 - Radial distribution functions $g(r)$ describing $H_2O - H_2O$ (green) correlations in the $CCPhe^{1:3}$ system under different hydration conditions: (a) 5%, (b) 10%, (c) 15%, (d) 20%, (e) 25%, and (f) 30% H_2O.

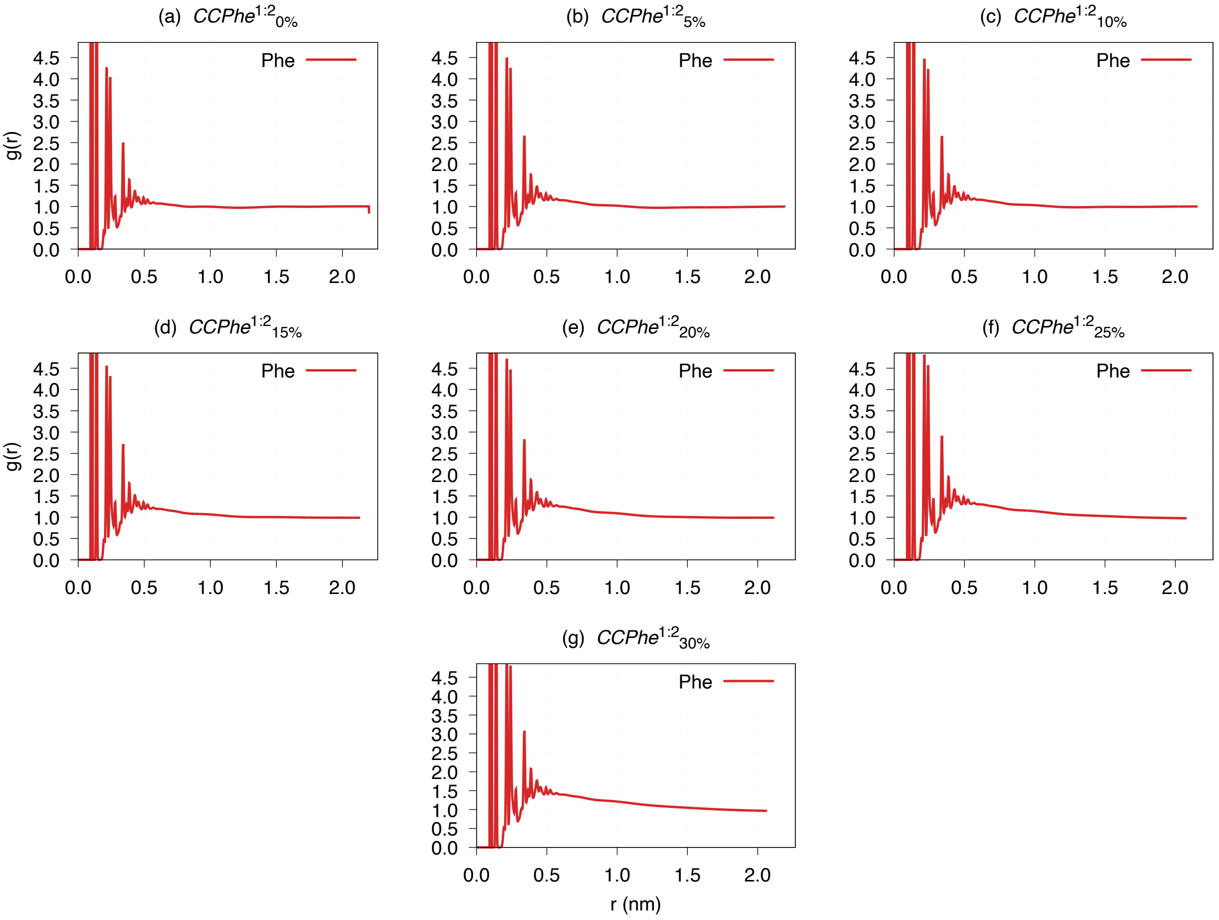

Figure S12 - Radial distribution functions $g(r)$ describing $Phe - Phe$ (red) correlations in the $CCPhe^{1:3}$ system under different hydration conditions: (a) pure, (b) 5%, (c) 10%, (d) 15%, (e) 20%, (f) 25%, and (g) 30% H_2O.

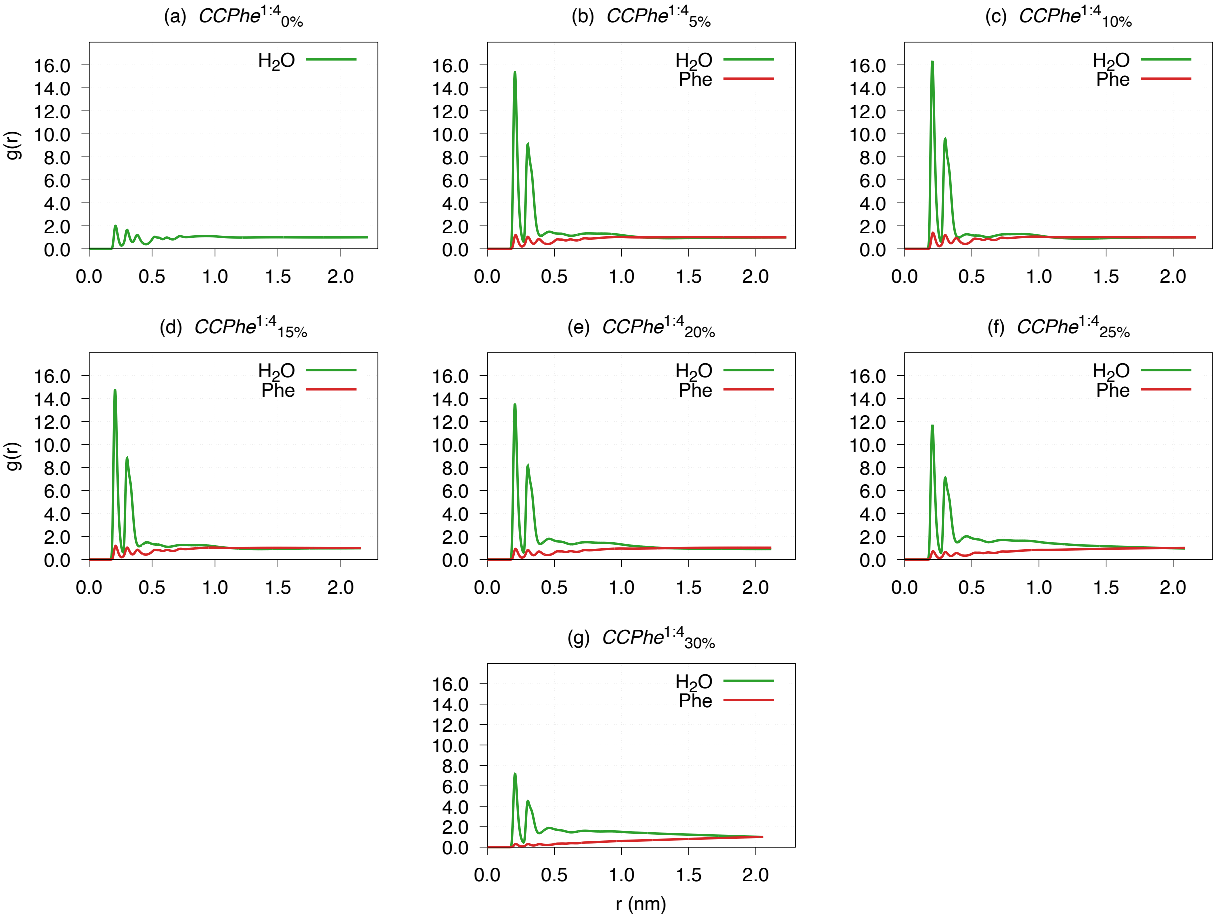

Figure S13 - Radial distribution functions $g(r)$ describing $Cl^{-} - H_2O$ (green) and $Cl^{-} - Phe$ (red) correlations in the $CCPhe^{1:4}$ system under different hydration conditions: (a) pure, (b) 5%, (c) 10%, (d) 15%, (e) 20%, (f) 25%, and (g) 30% H_2O.

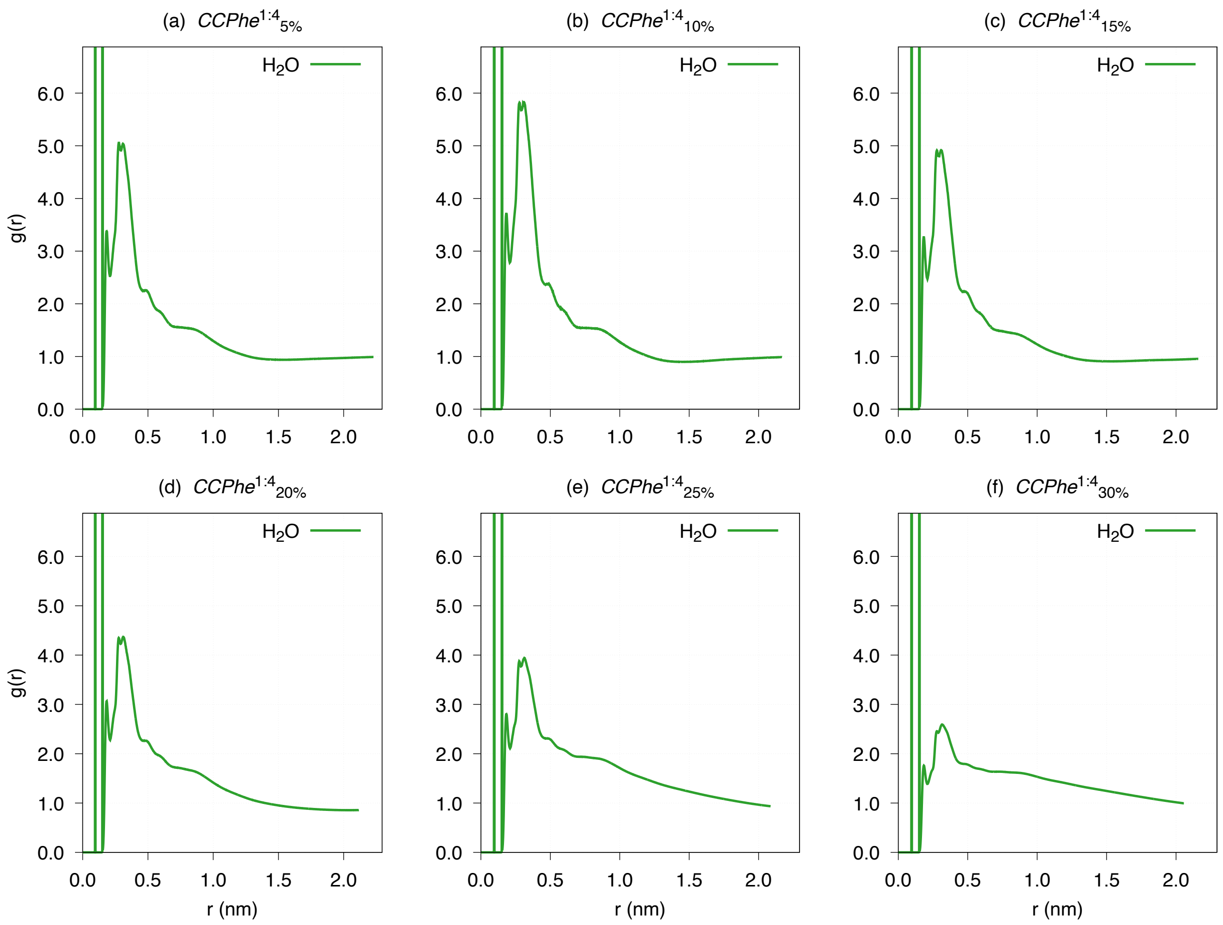

Figure S14 - Radial distribution functions $g(r)$ describing $H_2O - H_2O$ (green) correlations in the $CCPhe^{1:4}$ system under different hydration conditions: (a) 5%, (b) 10%, (c) 15%, (d) 20%, (e) 25%, and (f) 30% H_2O.

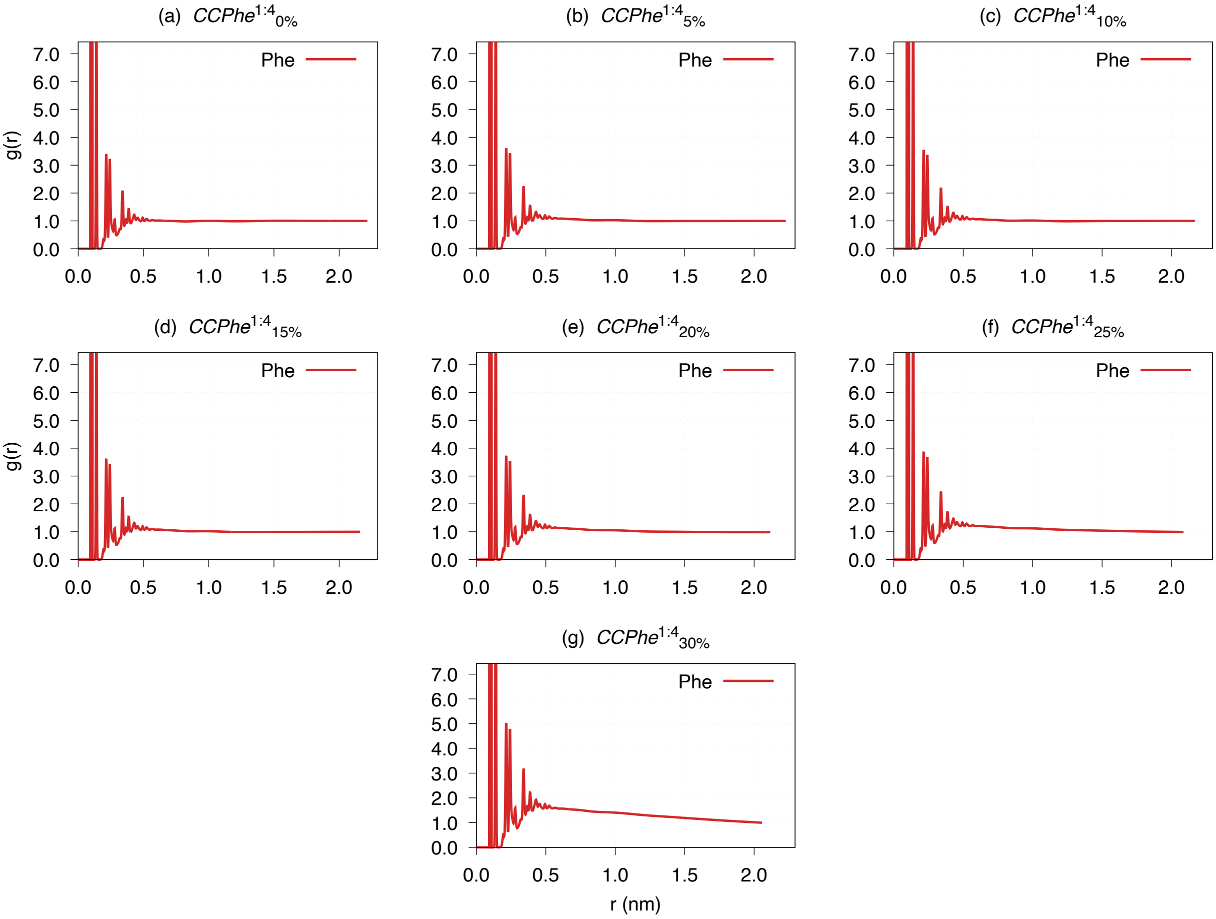

Figure S15 - Radial distribution functions $g(r)$ describing $Phe - Phe$ (red) correlations in the $CCPhe^{1:4}$ system under different hydration conditions: (a) pure, (b) 5%, (c) 10%, (d) 15%, (e) 20%, (f) 25%, and (g) 30% H_2O.
